# Characterization of Yak Common Biofluids Metabolome by Means of Proton Nuclear Magnetic Resonance Spectroscopy

**DOI:** 10.3390/metabo9030041

**Published:** 2019-03-02

**Authors:** Chenglin Zhu, Cheng Li, Yaning Wang, Luca Laghi

**Affiliations:** 1 Department of Agro-Food Science and Technology, University of Bologna, Piazza Goidanich 60, 47521 Cesena, Italy; chenglin.zhu2@unibo.it (C.Z.); yaning.wang@studio.unibo.it (Y.W.); 2 College of Food, Sichuan Agricultural University, Ya’an 625014, China; lichenglcp@163.com

**Keywords:** yak, *Bos grunniens*, serum, feces, urine, metabolomics, ^1^H-NMR

## Abstract

The aim of this study was to evaluate the metabolic profiles of yak (*Bos grunniens*) serum, feces, and urine by using proton nuclear magnetic resonance (^1^H-NMR), to serve as a reference guide for the healthy yak milieu. A total of 108 metabolites, giving information about diet, protein digestion, and energy generation or gut-microbial co-metabolism, were assigned across the three biological matrices. A core metabolome of 15 metabolites was ubiquitous across all biofluids. Lactate, acetate, and creatinine could be regarded as the most abundant metabolites in the metabolome of serum, feces, and urine, respectively. Metabolic pathway analysis showed that the molecules identified could be able to give thorough information about four main metabolic pathways, namely valine, leucine, and isoleucine biosynthesis; phenylalanine, tyrosine, and tryptophan biosynthesis; glutamine and glutamate metabolism; and taurine and hypotaurine metabolism.

## 1. Introduction

Metabolomics is a powerful approach to a biological system that aims to measure its low weight metabolites (<900 Da). When untargeted, this characterization of the metabolic phenotype provides holistic information on the system under investigation because it allows the study of its biochemical responses to intrinsic (genetics, protein expression) or environmental (diet, gut microbiota) stimuli [1]. 

^1^H-NMR spectroscopy is one of the main platforms for metabolomics because the very simple sample preparation and highly reproducible molecule quantification counterbalance a sensitivity lower than the one granted by other platforms such as mass spectrometry. For this reason, ^1^H-NMR spectroscopy has been employed in domestic animals to obtain the metabolite profiles of several biofluids, among which urine [2,3], serum [4,5,6], tracheal wash, and exhaled breath condensate [7]. 

The yak (*Bos grunniens*) is regarded as a very peculiar species of ruminant because it represents the main sustaining food source for the people who live in the region around the Himalayas, with an altitude ranging from 2500 to 5500 m with no frost-free periods, and mostly above the tree line. The genetic adaptive evolution of the yak to the harsh conditions has led to larger heart and lungs, and a higher erythrocyte count, compared to the cattle (*Bos taurus*) [8], in addition to a more efficient energy harvesting and nitrogen utilization [9,10]. Therefore, the yak is considered an ideal model animal for studying adaptation mechanisms to harsh conditions represented by low temperatures and a paucity of oxygen and energy sources. Despite these peculiarities, the physiology of yak has rarely been studied through an ”omics” approach. One reason for such a paucity of works may be the limited accessibility of the geographical areas where the yaks are bred, generally according to traditional practices. A few studies can be found focusing on yak milk [11,12,13] and its genome [8,14,15], with only one study that described the characteristics of yak meat [16]. The objective of the present study was to reduce this gap of information by characterizing, for the first time, the metabolome of yak serum, feces, and urine using ^1^H-NMR. This study is meant as a reference guide for researchers wishing to apply a metabolomics approach to the yak. For this reason, in compliance with the guidelines outlined by the consortium COSMOS (Coordination of Standards in Metabolomics; http://cosmos-fp7.eu/), the data have been made available on the open platform MetaboLights (study identifier: MTBLS841).

## 2. Materials and Methods 

### 2.1. Sampling of Biofluids

Serum, feces, and urine sampling was carried out in a public abattoir located in the pastoral area of Litang County (altitude 4000 m) at the end of August. This is the time of the year when yaks are traditionally slaughtered for meat, due to their optimal health conditions and highest weight. 

Five specimens of male Jiulong yak (approximately three years of age, receiving no supplementation) were randomly selected for the current experiment. The yaks were transported to the abattoir (without modern slaughtering equipment), held in lairage for 24 h (water supplementation until 3 h before slaughtering), and then sacrificed according to the local traditional manual procedures of yak slaughter [17].

Upon slaughtering, 10 mL of blood was collected using disposable syringes (10 mL, without clotting activator, Jiangyin Fanmei Medical Device Co., Ltd., Jiangyin, Jiangsu, China) from the abdominal vein and immediately transferred to sterile conical tubes. Blood samples were left at room temperature for 45 min to allow coagulation without centrifugation, and subsequently, the serum was separated from the blood clot. Urine samples were collected by cystocentesis upon direct bladder visualization using a sterile syringe while feces were collected as the animals defecated during the slaughtering process. All the above-mentioned samples were transported in dry ice and stored at −80 °C until analysis.

### 2.2. Metabolomics Analysis of Biofluids

We created an NMR analysis solution with 3-(trimethylsilyl)-propionic-2,2,3,3-d4 acid sodium salt (TSP) 10 mM in D_2_O, set at pH 7.00 ± 0.02 by means of 1 M phosphate buffer, containing also 10 μL of NaN_3_ 2 mM. TSP was employed as an NMR chemical-shift reference, while NaN_3_ avoided microbial proliferation.

Serum samples were prepared for ^1^H-NMR by thawing and centrifuging 1 mL of each sample for 15 min at 18,630 g and 4°C. 500 μL of supernatant was added to 100 μL of NMR analysis solution. Urine samples were prepared for ^1^H-NMR by means of thawing and centrifuging them for 15 min at 18,630 g at 4°C. An amount of supernatant equal to 350 μL was added to 350 μL of bi-distilled water and to 200 μL of NMR analysis solution. Fecal samples were prepared for ^1^H-NMR analysis by vortex mixing for 5 min 80 mg of stool with 1 mL of deionized water. The obtained mixes were then centrifuged for 15 min at 18,630 *g* and 4 °C, and 700 μL of supernatant was added to 200 μL of NMR analysis solution. Finally, each of the obtained samples was centrifuged again at the above conditions just before analysis.

^1^H-NMR spectra were recorded at 298 K with an AVANCE III spectrometer (Bruker, Milan, Italy) operating at a frequency of 600.13 MHz, equipped with the software Topspin 3.5. Following Zhu et al. [3]. The signals from broad resonances originating from large molecules were suppressed by a CPMG (Carr. Purcell. Meiboom. Gill) filter composed of 400 echoes with a τ of 400 μs and a 180° pulse of 24 μs, for a total filter of 330 ms. The water residual signal was suppressed by means of presaturation. This was done by employing the cpmgpr1d sequence, part of the standard pulse sequence library. Each spectrum was acquired by summing up 256 transients using 32 K data points over a 7184 Hz spectral window, with an acquisition time of 2.28 s. 

Differences in water and fibers content among samples were taken into consideration by probabilistic quotient normalization [18], more reliable than the once more common normalization on creatinine. Spectra phase was manually adjusted in Topspin, while the subsequent adjustments were performed in R computational language by means of script developed in-house [19]. After the removal of the residual water signal, ^1^H-NMR spectra were baseline-corrected by means of peak detection, according to the “rolling ball” principle [20], implemented in the baseline R package [21]. The signals were assigned by comparing their chemical shift and multiplicity with Chenomx software library (Chenomx Inc., Edmonton, Alberta, Canada, ver 8.3), as detailed in Figures S1–S36. 

In order to apply NMR as a quantitative technique [2], the recycle delay was set to 5 s by considering the relaxation time of the protons under investigation. Moreover, while TSP could be used as a reliable internal standard for urine and feces [22], the molecules of the first serum sample analyzed were quantified by means of an external standard, by taking advantage of the principle of reciprocity [23].

### 2.3. Pathway Analysis

Pathway analysis was performed using MetaboAnalyst 4.0 (https://www.metaboanalyst.ca) [24], which organizes the information about biochemical pathways described in KEGG database (https://www.genome.jp). In detail, pathway analysis used high-quality KEGG metabolic pathways as the backend knowledgebase. The Pathway Analysis module combined results from powerful pathway enrichment analysis with pathway topology analysis to identify the most relevant pathways involved in the conditions under study.

## 3. Results

### 3.1. ^1^H-NMR Spectra of Yak Serum, Feces, and Urine

**Figure 1:**
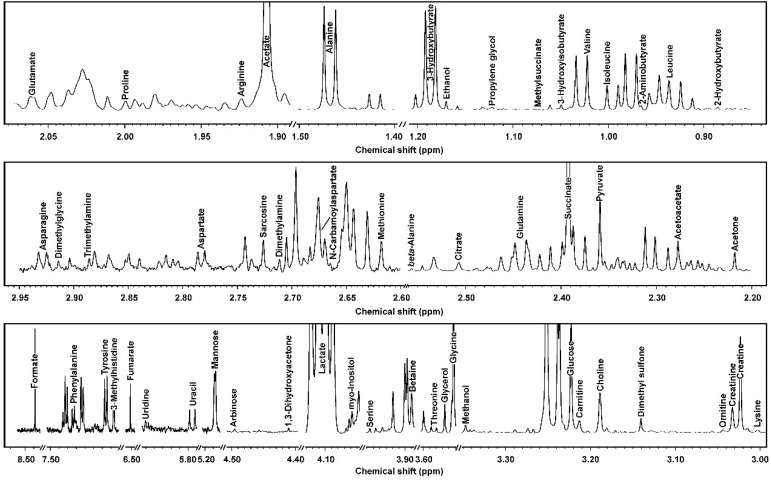
^1^H-NMR signals from yak serum, representative of those registered in the present work. The name of each molecule appears over the signal used for its quantification. To ease the reader’s visual inspection, for each portion the spectrum with a convenient signal-to-noise ratio has been selected.

**Figure 2:**
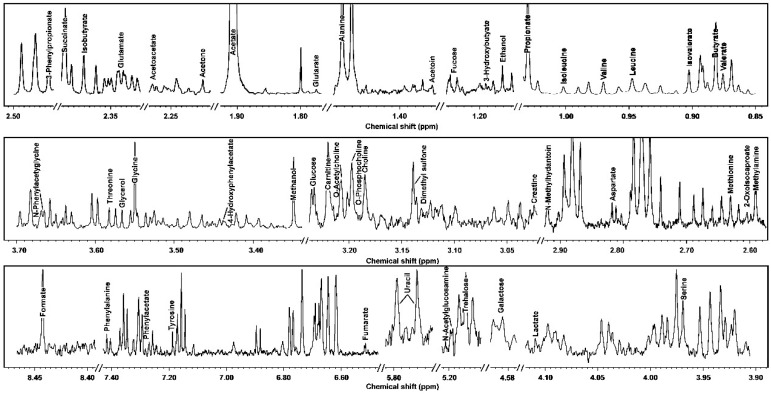
^1^H-NMR signals from yak feces, representative of those registered in the present work. The name of each molecule appears over the signal used for its quantification. To ease the reader’s visual inspection, for each portion the spectrum with a convenient signal-to-noise ratio has been selected.

**Figure 3:**
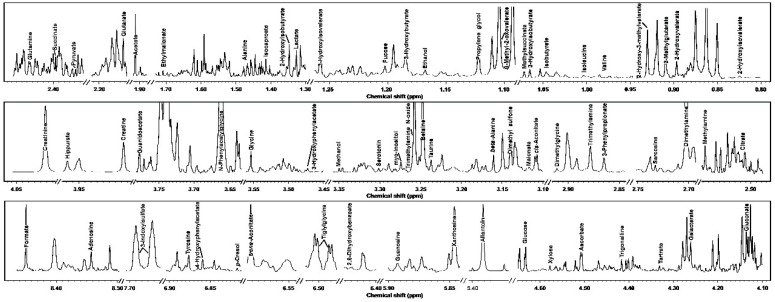
^1^H-NMR signals from yak urine, representative of those registered in the present work. The name of each molecule appears over the signal used for its quantification. To ease the reader’s visual inspection, for each portion the spectrum with a convenient signal-to-noise ratio has been selected.

In the current study, we were able to identify and quantify 109 molecules across yak serum, feces, and urine, giving information about diet, protein digestion, energy generation, or gut-microbial co-metabolism. For readability, Table 1 and Table 2 show the molecules that could be identified in all the biofluids, while the complete list is reported in Table S1. Typical ^1^H-NMR spectra of serum, feces, and urine are reported in Figure 1, Figure 2 and Figure 3, respectively.

**Figure 4:**
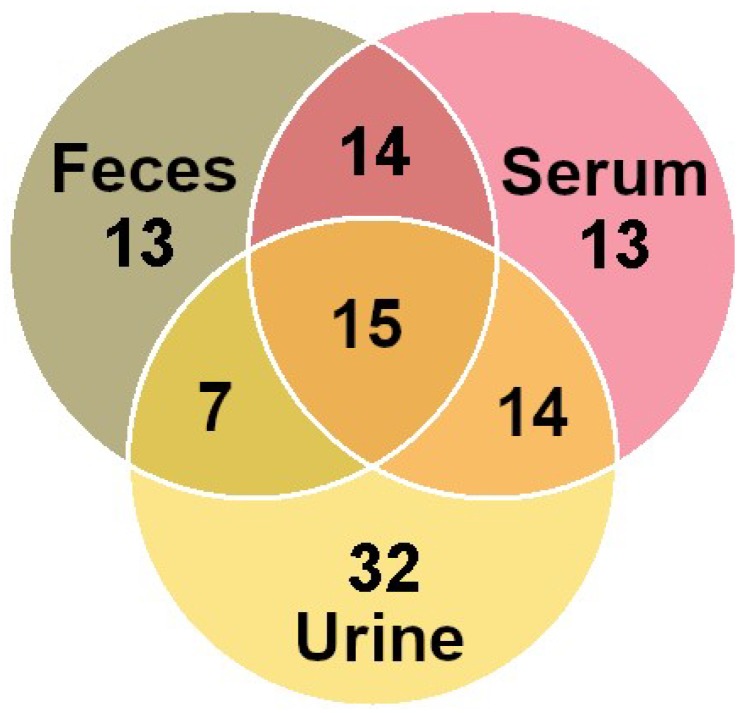
Venn diagram showing unique and shared metabolites among yak serum, urine, and feces. The number of metabolites is listed in each of the diagram components.

By means of ^1^H-NMR we were able to identify and quantify 56 molecules in serum, 49 in feces, and 68 in urine—nearly a twofold increase in comparison to previous works on cattle [4,26]. The area of the signals of the molecules overall assigned in serum, feces, and urine accounted on average for 85.04%, 87.04%, and 61.40% of the total spectral area, respectively. Among the molecules quantified, 15 were in common among the three biofluids, as shown in Figure 4.

### 3.2. Molecule Distribution by Class

**Figure 5:**
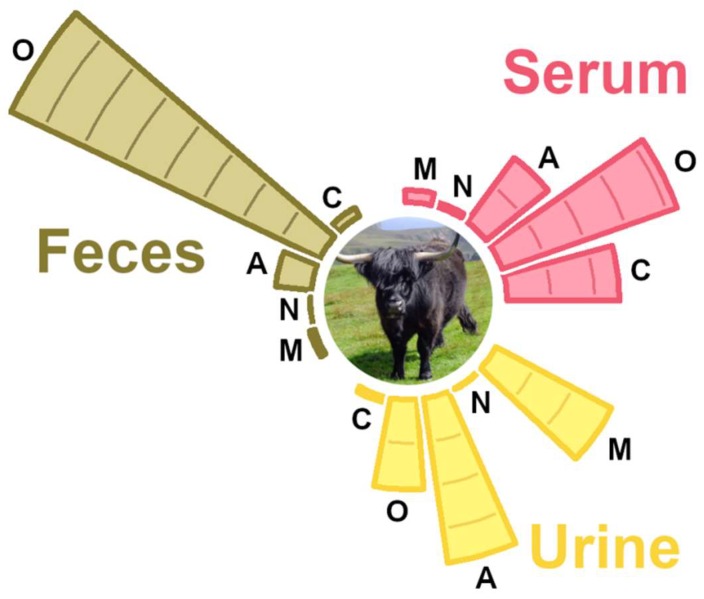
The relative abundance of the classes of molecules assigned in serum, feces, and urine metabolome. As a reference, the lines inside the bars are written in 10% steps. C = carbohydrates and derivatives, O = organic acids and derivatives, A = amino acids, peptides, and derivatives, N = nucleosides, nucleotides, and analogs, M = miscellaneous.

As shown in Figure 5, in serum and feces most of the molecules that were detected belong to the class of organic acids and their derivatives (47.09% and 86.92%, respectively), while amino acids, peptides, and analogs were mostly represented in the urine. As reported in detail in the supplementary material, lactate was the most concentrated molecule detected in serum (41.83%), followed by glucose (27.95%). In feces, the most concentrated molecule detected was acetate (49.04%), followed by propionate (17.58%) and butyrate (8.55%). In urine, the most concentrated substances detected were creatinine (28.69%), *N*-phenylacetylglycine (28.50%), and hippurate (13.28%).

### 3.3. Pathway Analysis

**Figure 6:**
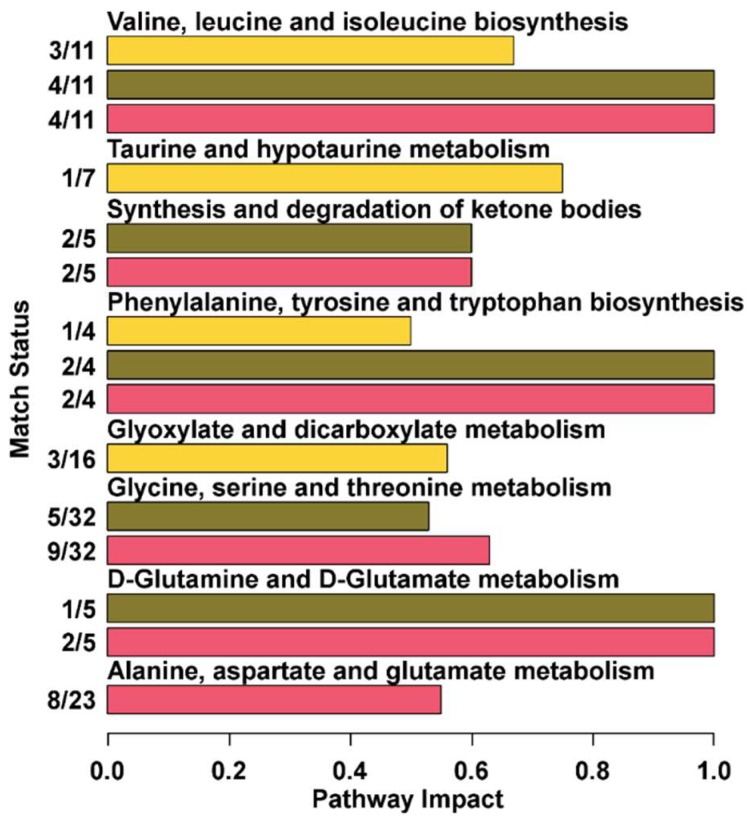
Metabolic pathways evidenced by enrichment analysis based on the metabolites detected in serum, feces, and urine (impact value > 0.5 [27]).

We wanted to understand which metabolic pathway could be described in sufficient detail by the molecules identified in the three biofluids studied. For this purpose, a pathway analysis was performed by means of the MetaboAnalyst platform on each biofluid, as detailed in Figure 6. Overall, three pathways were described with an impact as high as 1, namely valine, leucine, and isoleucine biosynthesis; phenylalanine, tyrosine, and tryptophan biosynthesis; and glutamine and glutamate metabolism. In addition, the first two were described in high detail throughout each of the biofluids studied. Five more pathways were described with an impact higher than 0.5. These pathways included alanine, aspartate, and glutamate metabolism; glycine, serine, and threonine metabolism; synthesis and degradation of ketone bodies; d-glutamine, glyoxylate, and dicarboxylate metabolism; and taurine and hypotaurine metabolism. 

## 4. Discussion

### 4.1. Yak Serum Metabolome

To our knowledge, the metabolome of yak serum has never been described before in literature. The metabolome of serum has been studied before in cattle, a closely related species, but only with a focus on the biomarkers of specific diseases or viruses, such as milk fever [5], footrot [6], *Mycobacterium tuberculosis* [4], and *Mycobacterium avium* subsp. *paratuberculosis* [28].

Lactate is the most abundant low-weight metabolite we were able to observe in the yak serum. In cattle, its presence has been mainly attributed to ruminal microflora [29]. Its presence in yak serum plays a special role, because its high concentration, together with pyruvate, is a direct consequence of the adaptation to low oxygen levels connected to altitude [30,31,32]. In fact, hypoxia prompts a shift towards anaerobic energy generation through an active withdrawal of pyruvate from the TCA cycle towards lactate production [33,34,35]. Another explanation for the presence of lactate in the serum samples is that the blood was collected after the slaughter of the animal, and so lactate could have been remarkably concentrated due to stress. Finally, the sample preparation did not include a centrifugation step, so the lactate may have also come from the erythrocytes that were damaged during freezing and thawing.

Glucose was the second most abundant molecule we quantified in yak serum. Experiments on rats suggest that this molecule could offer another direct way to follow the consequences of altitude on yak metabolism. Glucose utilization has been found to increase with altitude, because alternative energy generation pathways, such as those involving proteins, can be insufficient for the maintenance of glycemia, especially after exercise [31].

### 4.2. Yak Feces Metabolome

The metabolic composition of fecal extracts provides a window for elucidating the complex metabolic interplay between mammals and their intestinal ecosystem. Moreover, the metabolite profile can yield information on a range of gut conditions [36]. To the best of the authors’ knowledge, feces from cattle have been observed from the microbiome point of view [37], but never from a metabolomics perspective. Interestingly, considering the total concentration of the molecules detected in the feces equal to 100, 75.17% was represented by the short chain fatty acids (SCFAs) acetate, propionate, and butyrate. SCFAs are major products of the microbial fermentation of fiber polysaccharides in the rumen, which may play an important role in the efficient harvesting of energy from plants [38]. This efficiency seems to be mediated by ruminal microbiome selection, as recently observed by Zhang et al. [37], who compared animals that were genetically adapted and non-adapted to high altitudes. Notably, Zhang et al. also found that the rumen microbiome of high-altitude ruminants showed a significant up-regulation of amino acids metabolism. This finding could be coherent with our pathway analysis, where the most important pathways described by the yak feces metabolome indeed entail amino acid metabolism.

### 4.3. Yak Urine Metabolome

As plasma sampling is invasive by nature, and fecal extracts inherently vary in composition with the relative abundance of microbial–mammalian metabolites, urine is regarded as the most appropriate biofluid for the purposes of metabonomic analysis [25]. 

The most abundant metabolite we quantified in yak urine was creatinine. This molecule is synthesized in connection to the absorption of creatine phosphate by the muscles, then released to the serum, and cleared by kidneys. In cattle, this molecule has been found to be proportional to muscle activity, with specific reference to heart and respiration rates [39], but this is higher in yak than in cattle [8].

The second and third most abundant metabolites we quantified in yak urine were hippurate and *N*-phenylacetylglycine, which are regarded as urinary metabolomic biomarkers of the response to hypobaric hypoxia, according to Koundal et al. [40]. Koundal et al. built a mouse model to illustrate how mice urinary metabolomics were changing in response to hypobaric hypoxia. Taurine metabolism and TCA were highlighted as important pathways that might have contributed to hypobaric hypoxia-induced pathophysiology, which is in accordance with our findings. Hippurate and *N*-phenylacetylglycine are sorted as metabolites relating to gut microflora metabolism and they also demonstrated that lowered urinary hippurate and *N*-phenylacetylglycine indicate decreased gut microflora.

In ruminants, the purine derivative allantoin is regarded as a biomarker of nitrogen clearance, which is generated from uric acid by uricase. In response to the harsh forage environment, yaks expel through urine a lower amount of purine derivatives [9], with mechanisms to recycle nitrogen, which are probably linked to the reduced degradation of nucleic acids by rumen microbiota [41].

## 5. Conclusions

To the best of our knowledge, this is the first work where ^1^H-NMR has been employed to study yak biofluids from a metabolomics perspective. Due to the small number of samples investigated, the results presented here should be regarded as preliminary. Nevertheless, we were able to characterize as many as 56, 49, and 68 metabolites in serum, feces, and urine, respectively, which is almost two times more than previously reported for cattle. The most concentrated metabolites in the three biofluids were found related to some of the biological reasons for the adaptation of yak to the ecological niche represented by extreme altitudes. Future research focusing on the comparison between wild and captive yaks could be useful to even better characterize the metabolome of the yak.

## Figures and Tables

**Table 1:** Metabolites identified by ^1^H-NMR in common among yak serum, feces, and urine.

Molecule	ppm *	Functional Group	Multiplicity **	Source [25] ***
3-Hydroxybutyrate	1.1863	CH_3_	d	E
Acetate	1.9071	CH_3_	s	P
Alanine	1.4675	CH_3_	d	P
Creatine	3.0222	CH_2_	s	P
Dimethyl sulfone	3.1391	CH_3_	s	D, M
Ethanol	1.1699	CH_3_	t	E, M
Formate	8.4446	CH	s	E
Glucose	3.2233	CH-2	dd	D, E
Glycine	3.5533	CH_2_	s	P
Isoleucine	1.0020	CH_3_-9	d	P
Lactate	4.1059	CH	dd	E
Methanol	3.3481	CH_3_	s	E
Succinate	2.3933	CH_2_	s	P, E
Tyrosine	7.1776	CH-3	d	P
Valine	1.0206	CH_3_-7	d	P

* Chemical shift of the signal employed for quantification; ** Splitting pattern of the signal (s = singlet; d = doublet; t = triplet; dd = doublet of doublets); *** Main source of the molecules (D = dietary metabolites, P = protein and amino acid metabolism, E = energy metabolism, M = gut-microbial co-metabolism).

**Table 2:** Concentration (median and interquartile range) of metabolites identified by ^1^H-NMR in common among yak serum, feces, and urine

Molecules	Serum (mmol/L)	Feces (mmol/g)	Urine (mmol/L)
3-Hydroxybutyrate	1.40 × 10^−1^ (3.56 × 10^−2^)	1.52 × 10^−5^ (1.30 × 10^−5^)	1.28 × 10^−3^ (7.54 × 10^−4^)
Acetate	1.40 × 10^−1^ (1.59 × 10^−1^)	3.66 × 10^−2^ (1.06 × 10^−2^)	5.66 × 10^−4^ (4.03 × 10^−4^)
Alanine	2.78 × 10^−1^ (1.27 × 10^−2^)	5.16 × 10^−4^ (3.25 × 10^−4^)	2.14 × 10^−4^ (4.23 × 10^−5^)
Creatine	2.01 × 10^−1^ (2.12 × 10^−1^)	1.91 × 10^−5^ (1.88 × 10^−5^)	4.00 × 10^−2^ (1.80 × 10^−2^)
Dimethyl sulfone	1.23 × 10^−2^ (4.15 × 10^−3^)	1.29 × 10^−5^ (1.07 × 10^−5^)	6.15 × 10^−4^ (1.40 × 10^−4^)
Ethanol	4.69 × 10^−3^ (2.74 × 10^−3^)	7.15 × 10^−5^ (2.98 × 10^−5^)	2.62 × 10^−4^ (3.81 × 10^−5^)
Formate	1.78 × 10^−2^ (5.69 × 10^−3^)	1.17 × 10^−4^ (2.43 × 10^−5^)	2.50 × 10^−4^ (1.70 × 10^−4^)
Glucose	1.37 (5.97 × 10^−1^)	3.51 × 10^−4^ (5.47 × 10^−5^)	8.33 × 10^−4^ (4.09 × 10^−4^)
Glycine	5.11 × 10^−1^ (3.91 × 10^−1^)	1.77 × 10^−4^ (7.46 × 10^−6^)	8.52 × 10^−4^ (5.64 × 10^−4^)
Isoleucine	4.35 × 10^−2^ (1.62 × 10^−2^)	6.20 × 10^−5^ (1.39 × 10^−4^)	1.33 × 10^−4^ (7.24 × 10^−5^)
Lactate	7.25 (5.46 × 10^−1^)	5.57 × 10^−5^ (5.13 × 10^−5^)	9.37 × 10^−4^ (1.87 × 10^−4^)
Methanol	7.14 × 10^−3^ (1.78 × 10^−3^)	9.12 × 10^−5^ (4.36 × 10^−5^)	3.95 × 10^−5^ (2.32 × 10^−5^)
Succinate	2.01 × 10^−1^ (4.09 × 10^−2^)	1.12 × 10^−4^ (4.47 × 10^−5^)	9.15 × 10^−5^ (7.46 × 10^−5^)
Tyrosine	2.53 × 10^−2^ (1.22 × 10^−2^)	1.16 × 10^−4^ (6.81 × 10^−5^)	1.23 × 10^−3^ (2.26 × 10^−5^)
Valine	1.48 × 10^−1^ (1.14 × 10^−2^)	1.91 × 10^−4^ (5.42 × 10^−5^)	1.22 × 10^−4^ (3.45 × 10^−5^)

## Supplementary Materials

The following are available online at https://www.mdpi.com/2218-1989/9/3/41/s1, Figures: Supplemental_Material.docx, Tables: Supplemental_Material.docx and Supplemental_Material.xlsx
