# Supplementary material for: Characterization of Yak Common Biofluids Metabolome by Means of Proton Nuclear Magnetic Resonance Spectroscopy
Source: Metabolites. 2019 Mar 2;9(3):41. doi: 10.3390/metabo9030041 (PMC6468419; doi:10.3390/metabo9030041)
Supplement: Supplementary file 1 [file metabolites-09-00041-s001.zip › metabolites-09-00041-s001/Supplemental_Material.pdf]

Characterization of yak common biofluids metabolome by means of proton nuclear magnetic resonance spectroscopy

Chenglin Zhu, Cheng Li, Yaning Wang, Luca Laghi

SUPPLEMENTAL MATERIAL

Table S1: Metabolites identified by ^{1}H-NMR in yak biofluids.

Molecules	ppm	Functional group	Multiplicity	Molecule source	Groups
1,3-Dihydroxyacetone	4.4122	CH_2	s	D	Serum
2,6-Dihydroxybenzoate	6.4221	CH-2	d	D	Urine
2-Aminobutyrate	0.9648	CH_3	t	P	Serum
2-Hydroxy-3-methylvalerate	0.9310	CH_3-9	d	P	Urine
2-Hydroxybutyrate	0.8859	CH_3	t	P	Serum
2-Hydroxyisobutyrate	1.3478	CH_3	s	D	Urine
2-Hydroxyisovalerate	0.8244	CH_3	d	P	Urine
2-Hydroxyvalerate	0.8970	CH_3	t	E	Urine
2-Oxoisocaproate	2.6038	CH_2	d	P	Feces
3-Hydroxybutyrate	1.1863	CH_3	d	E	Serum, Urine, Feces
3-Hydroxyisobutyrate	1.0503	CH_3	d	P	Serum, Urine
3-Hydroxyisovalerate	1.2585	CH_3	s	P	Urine
3-Hydroxyphenylacetate	3.4718	CH_2	s	P	Urine
3-Indoxylsulfate	7.6894	CH-6	d	D, P	Urine
3-Methyl-2-oxovalerate	1.1042	CH3-9	d	P	Urine
3-Methylglutarate	0.9095	CH_3	d	P	Urine
3-Methylhistidine	7.1366	CH-5	s	P	Serum
3-Phenylpropionate	2.8659	CH_2-7	m	p	Urine, Feces
4-Hydroxyphenylacetate	6.8582	CH-3	d	D	Urine, Feces
Acetate	1.9071	CH_3	s	P	Serum, Urine, Feces
Acetoacetate	2.2688	CH_3	s	E	Serum, Feces
Acetoin	1.3583	CH_3-3	d	E	Feces
Acetone	2.2178	CH_3	s	E	Serum, Feces
Adenosine	8.3436	CH-7	s	P	Urine
Alanine	1.4675	CH_3	d	P	Serum, Urine, Feces
Allantoin	5.3643	CH	s	P, M	Urine
Arabinose	4.5038	CH-2	d	M, E	Serum
Arginine	1.9229	CH_2-6	m	P	Serum

Ascorbate	4.5072	CH	d	D, E	Urine
Asparagine	2.9282	CH_2	dd	P	Serum
Aspartate	2.7842	CH_2	dd	P	Serum, Feces
beta-Alanine	2.5558	CH_2-4	t	P	Serum, Urine
Betaine	3.8894	CH_2	s	P	Serum, Urine
Butyrate	0.8818	CH_3	t	E	Feces
Carnitine	3.2208	CH_3	s	E	Serum, Feces
Choline	3.1888	CH_3	s	E, P	Serum, Feces
cis-Aconitate	3.1089	CH_2	d	E	Urine
Citrate	2.5093	CH_2	d	P	Serum, Urine
Creatine	3.0222	CH_2	s	P	Serum, Urine, Feces
Creatinine	3.0325	CH_3	s	P	Serum, Urine
Dimethyl sulfone	3.1391	CH_3	s	D, M	Serum, Urine, Feces
Dimethylamine	2.7117	CH_3	s	P	Serum, Urine
Dimethylglycine	2.9124	CH_3	s	E, P	Serum, Urine
Ethanol	1.1699	CH_3	t	E, M	Serum, Urine, Feces
Ethylmalonate	1.7091	CH_2	m	E	Urine
Formate	8.4446	CH	s	E	Serum, Urine, Feces
Fucose	1.2024	CH_3	d	E	Urine, Feces
Fumarate	6.5090	CH-5	s	E	Serum, Feces
Galactarate	4.2597	CH	s	E	Urine
Galactose	4.5825	CH-2	d	E	Feces
Gluconate	4.1362	CH-9	d	E	Urine
Glucose	3.2233	CH-2	dd	D, E	Serum, Urine, Feces
Glutamate	2.0606	CH_2-6	m	P	Serum, Feces
Glutamine	2.4430	CH	m	P	Serum, Urine
Glutarate	2.1540	CH_2	t	E, P	Urine, Feces
Glycerol	3.5664	CH_2	dd	E	Serum, Feces
Glycine	3.5533	CH_2	s	P	Serum, Urine, Feces
Guanidoacetate	3.7782	CH_2	s	P	Urine
Guanosine	5.8929	CH-2	d	P	Urine
Hippurate	3.9607	CH_2	d	P	Urine
Isobutyrate	1.0473	CH_3	d	M	Urine, Feces
Isocaproate	1.4133	CH_2-5	m	E	Urine
Isoleucine	1.0020	CH_3-9	d	P	Serum, Urine, Feces
Isovalerate	0.9030	CH_3	d	P	Feces
Lactate	4.1059	CH	dd	E	Serum, Urine, Feces
Leucine	0.9356	CH_3-9	d	P	Serum, Feces
Lysine	3.0077	CH_2-9	t	P	Serum
Malonate	3.1199	CH_2	s	D, E	Urine
Mannose	5.1767	CH-6	d	E	Serum
Methanol	3.3481	CH_3	s	E	Serum, Urine, Feces
Methionine	2.6178	CH_2-3	t	P	Serum, Feces

Methylamine	2.5878	CH_3	s	D	Urine, Feces
Methylsuccinate	1.0742	CH_3	d	P, E	Serum, Urine
myo-Inositol	4.0585	CH-2	t	E	Serum, Urine
N-Acetylglucosamine	5.1989	CH	d	E, P	Feces
N-Carbamoylaspartate	2.6698	CH_2	dd	P	Serum
N-Methylhydantoin	2.9129	CH_3	s	P	Feces
N-Phenylacetylglycine	3.6605	CH_2-11	s	P	Urine, Feces
O-Acetylcholine	3.2081	CH_3	s	E	Feces
O-Phosphocholine	3.1977	CH_3	s	E	Feces
Ornithine	3.0440	CH_2-8	t	P	Serum
p-Cresol	6.8170	CH-2	d	P	Urine
Phenylacetate	7.2826	CH-4	m	P	Feces
Phenylalanine	7.3669	CH-4	t	P	Serum, Feces
Proline	1.9998	CH_2-3	m	P	Serum
Propionate	1.0292	CH_3	t	P	Feces
Propylene glycol	1.1227	CH_3	d	E	Serum, Urine
Pyruvate	2.3573	CH_3	s	E	Serum, Urine
Sarcosine	2.7262	CH_3	s	P	Serum, Urine
Serine	3.9555	CH_2	dd	P	Serum, Feces
Serotonin	3.3023	CH_2	t	P	Urine
Succinate	2.3933	CH_2	s	P, E	Serum, Urine, Feces
Tartrate	4.3311	CH	s	D	Urine
Taurine	3.2368	CH_2-6	t	P	Urine
Threonine	3.5838	CH-4	d	P	Serum, Feces
Tiglylglycine	6.4968	CH	m	P	Urine
trans-Aconitate	6.5814	CH	s	E	Urine
Trehalose	5.1837	CH	s	E	Feces
Trigonelline	4.4165	CH_3	s	D	Urine
Trimethylamine	2.8226	CH_3	s	D	Serum, Urine
Trimethylamine N-oxide	3.2589	CH_3	s	D	Urine
Tyrosine	7.1776	CH-3	d	P	Serum, Urine, Feces
Uracil	5.7903	CH-5	d	P	Serum, Feces
Uridine	5.9079	CH-2	d	P	Serum
Valerate	0.8756	CH_3	t	D	Feces
Valine	1.0206	CH_3-7	d	P	Serum, Urine, Feces
Xanthosine	5.8460	CH-2	d	P	Urine
Xylose	4.5781	CH-2	d	E	Urine

Molecules assignment and quantification

Figures S1-S36: Pictorial description of the molecules assignment procedure by Chenomx software. Upper panel - portions of the representative spectrum. Lower panel - The spectrum (black line) superimposed to the simulated signals (red line) for each of the molecules listed. The line connecting each name with the spectra highlights the signal used for quantification purposes.

Yak Serum

1

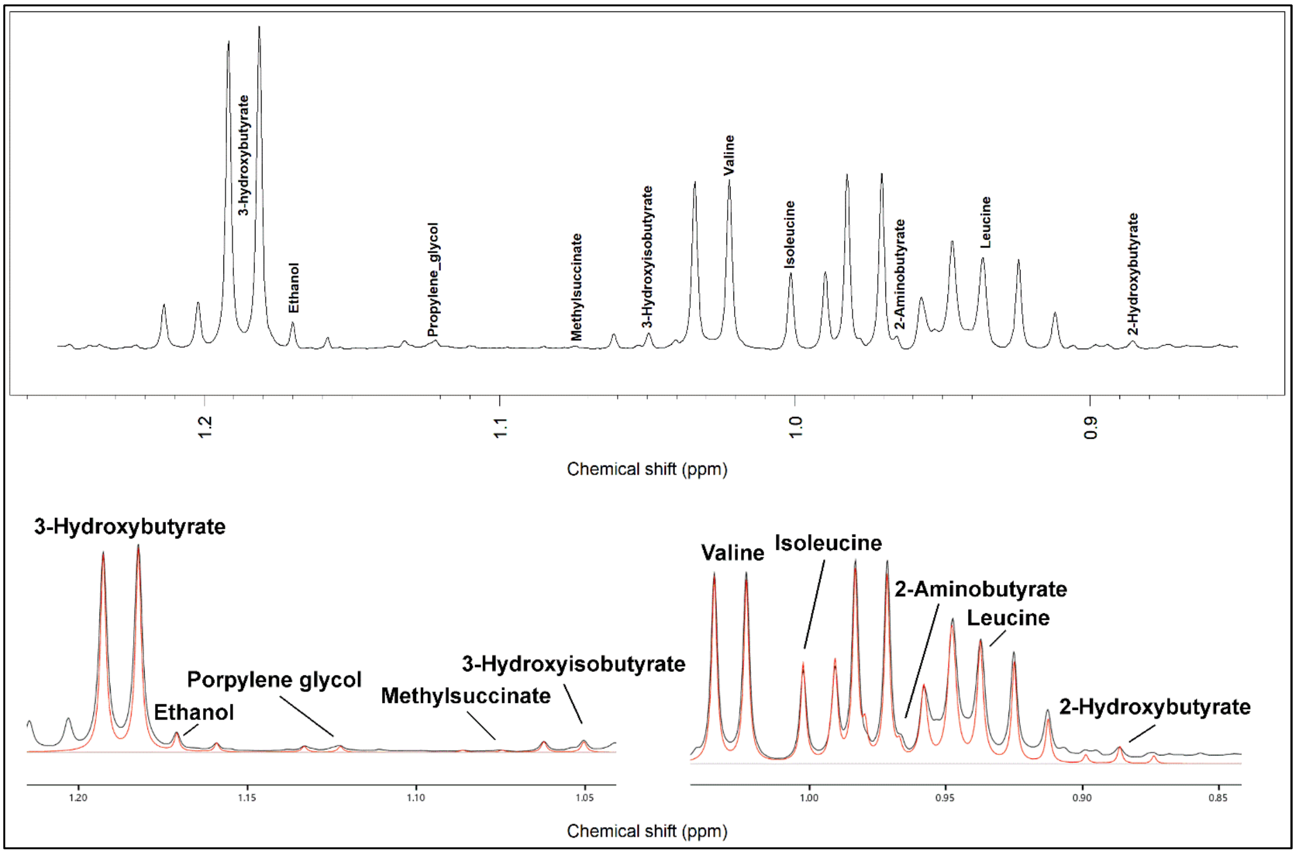

2

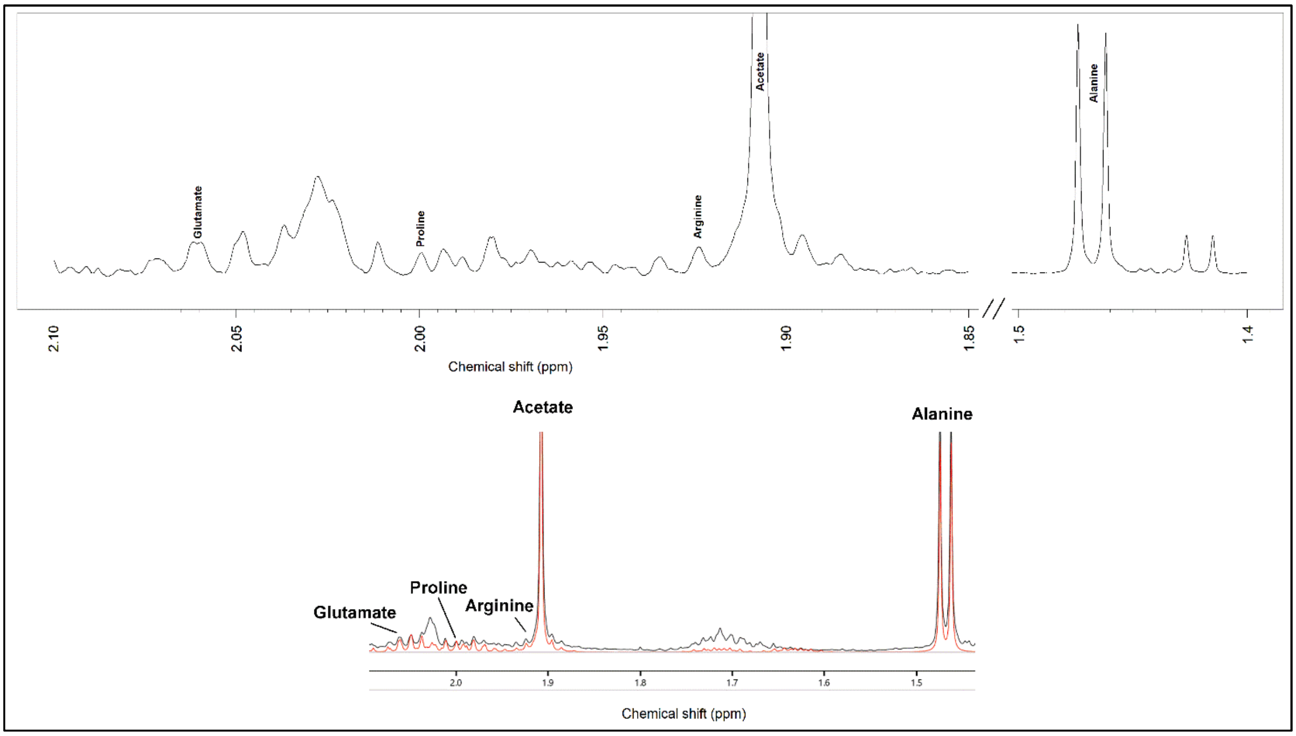
Glutamate
Proline
Arginine
Acetate
Alanine
2.10
2.05
2.00
1.95
1.90
1.85
1.5
1.4
Chemical shift (ppm)
Acetate
Alanine
Proline
Glutamate
Arginine
2.0
1.9
1.8
1.7
1.6
1.5
Chemical shift (ppm)

3

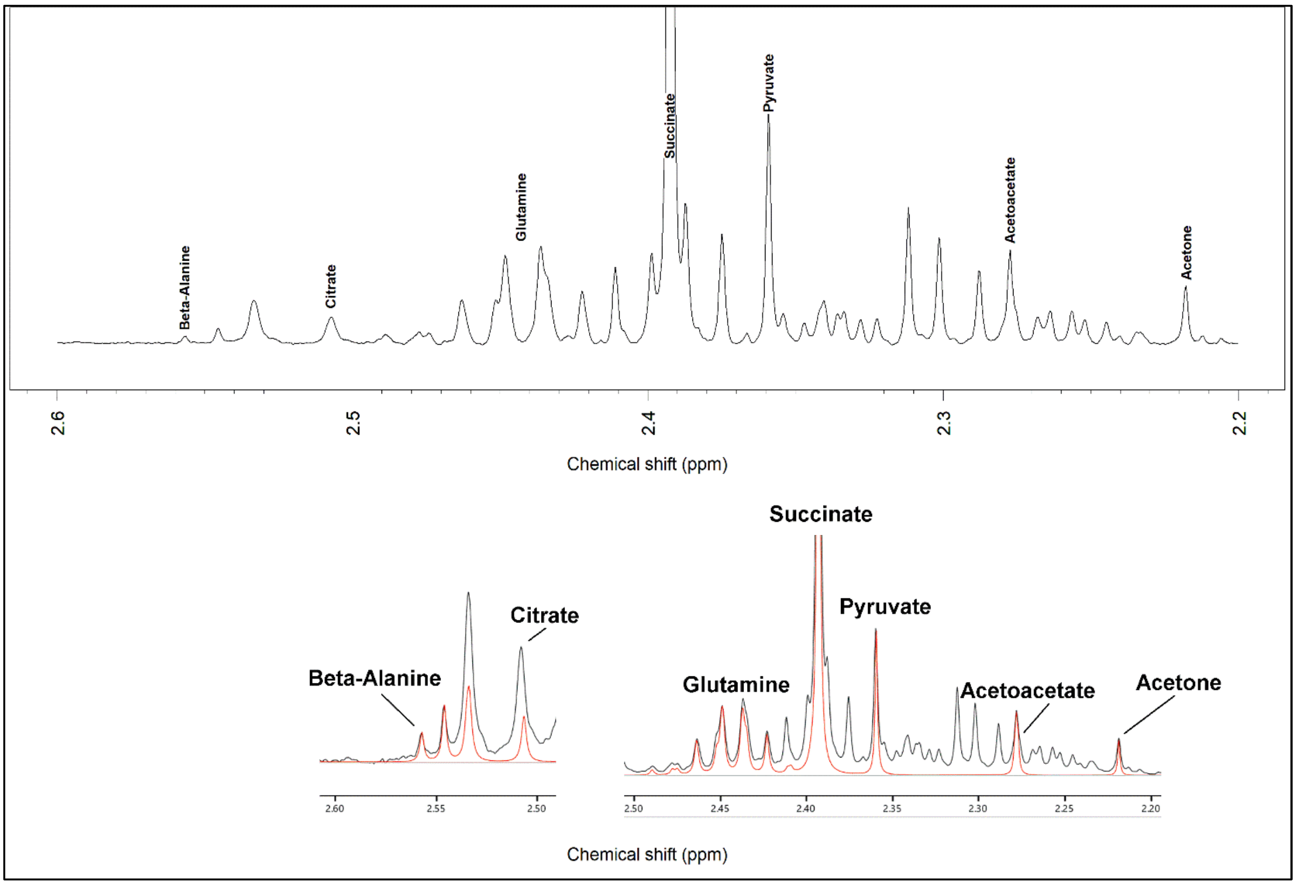
Beta-Alanine
Citrate
Glutamine
Succinate
Pyruvate
Acetoacetate
Acetone
2.6
2.5
2.4
2.3
2.2
Chemical shift (ppm)
Succinate
Pyruvate
Citrate
Beta-Alanine
Glutamine
Acetoacetate
Acetone
2.60
2.55
2.50
2.50
2.45
2.40
2.35
2.30
2.25
2.20
Chemical shift (ppm)

4

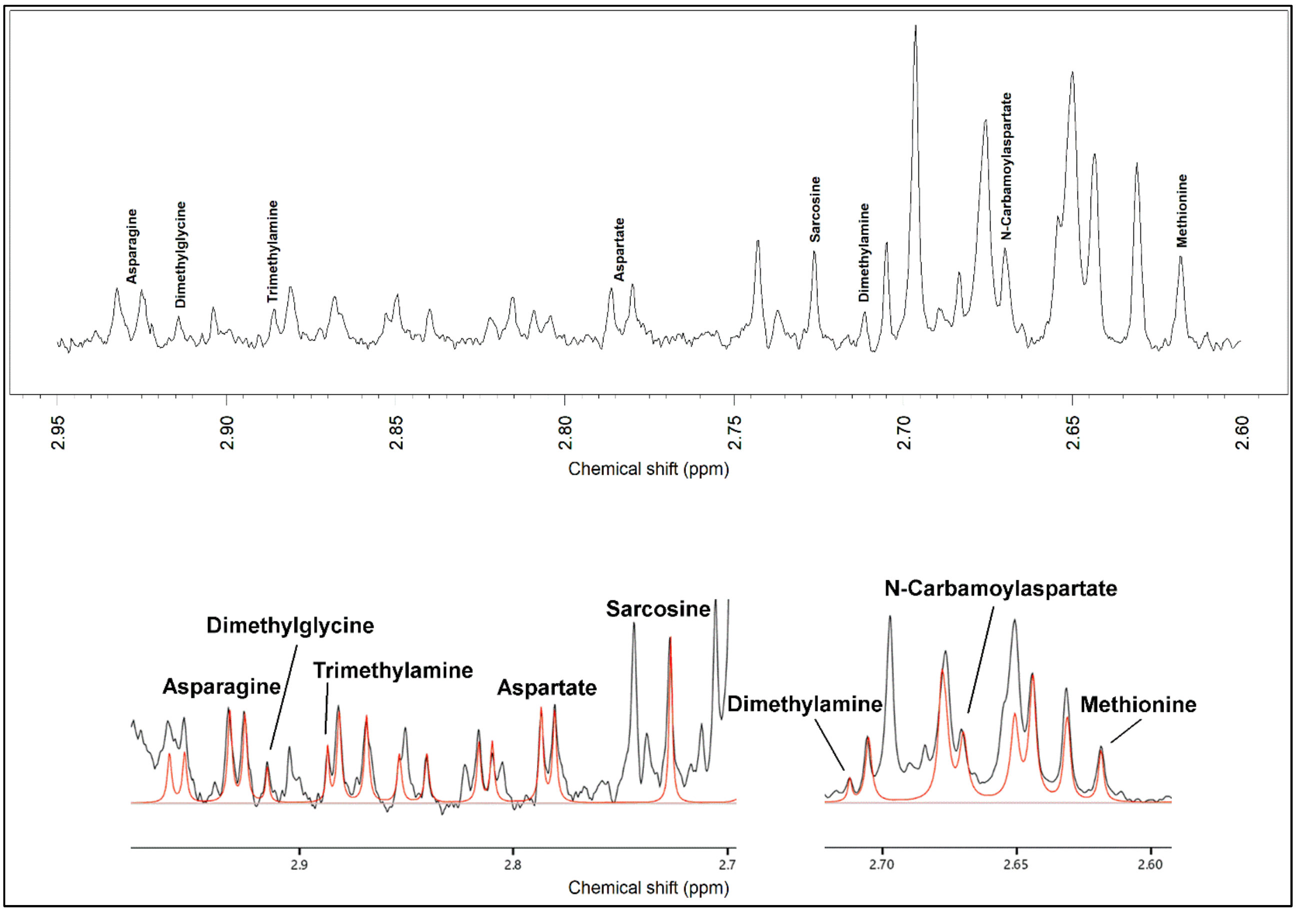
Asparagine
Dimethylglycine
Trimethylamine
Aspartate
Sarcosine
Dimethylamine
N-Carbamoylaspartate
Methionine
2.95
2.90
2.85
2.80
2.75
2.70
2.65
2.60
Chemical shift (ppm)
Dimethylglycine
Asparagine
Trimethylamine
Aspartate
Sarcosine
N-Carbamoylaspartate
Dimethylamine
Methionine
2.9
2.8
2.7
2.70
2.65
2.60
Chemical shift (ppm)

5

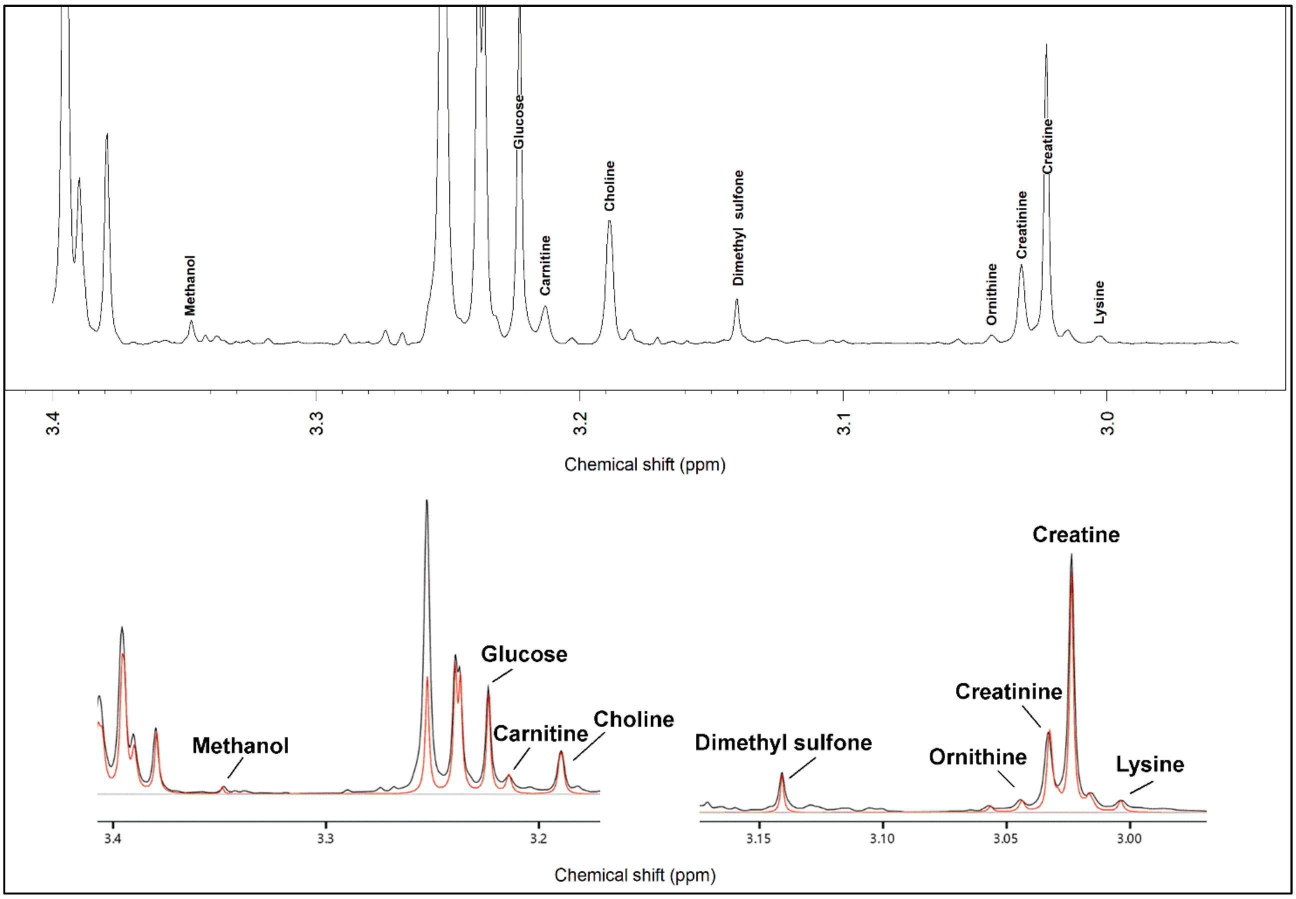
Methanol
Glucose
Carnitine
Choline
Dimethyl sulfone
Ornithine
Creatinine
Creatine
Lysine
3.4
3.3
3.2
3.1
3.0
Chemical shift (ppm)
Creatine
Glucose
Creatinine
Choline
Carnitine
Methanol
Dimethyl sulfone
Ornithine
Lysine
3.4
3.3
3.2
3.15
3.10
3.05
3.00
Chemical shift (ppm)

6

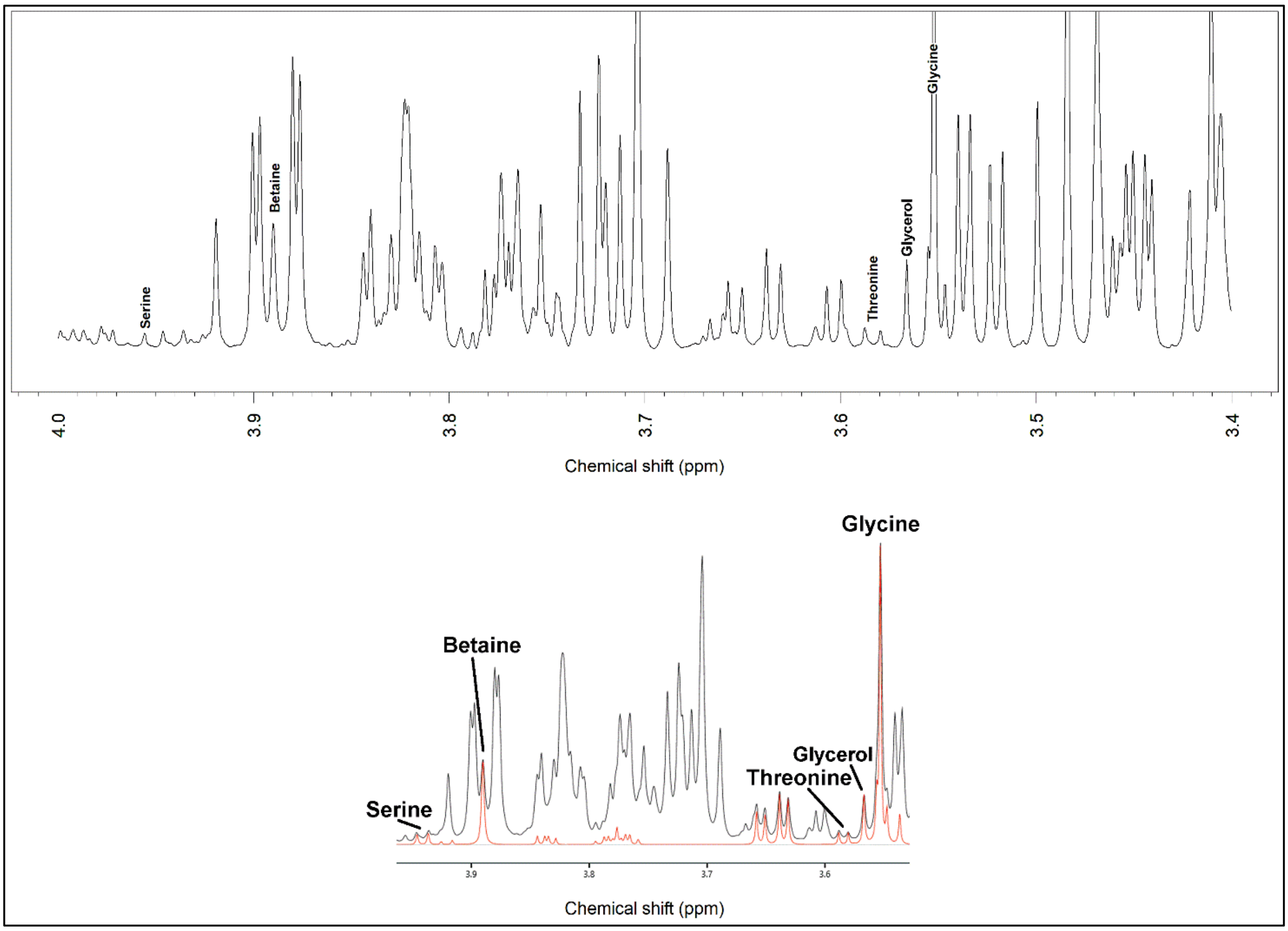
Serine
Betaine
Threonine
Glycerol
Glycine
4.0
3.9
3.8
3.7
3.6
3.5
3.4
Chemical shift (ppm)
Glycine
Betaine
Glycerol
Threonine
Serine
3.9
3.8
3.7
3.6
Chemical shift (ppm)

7

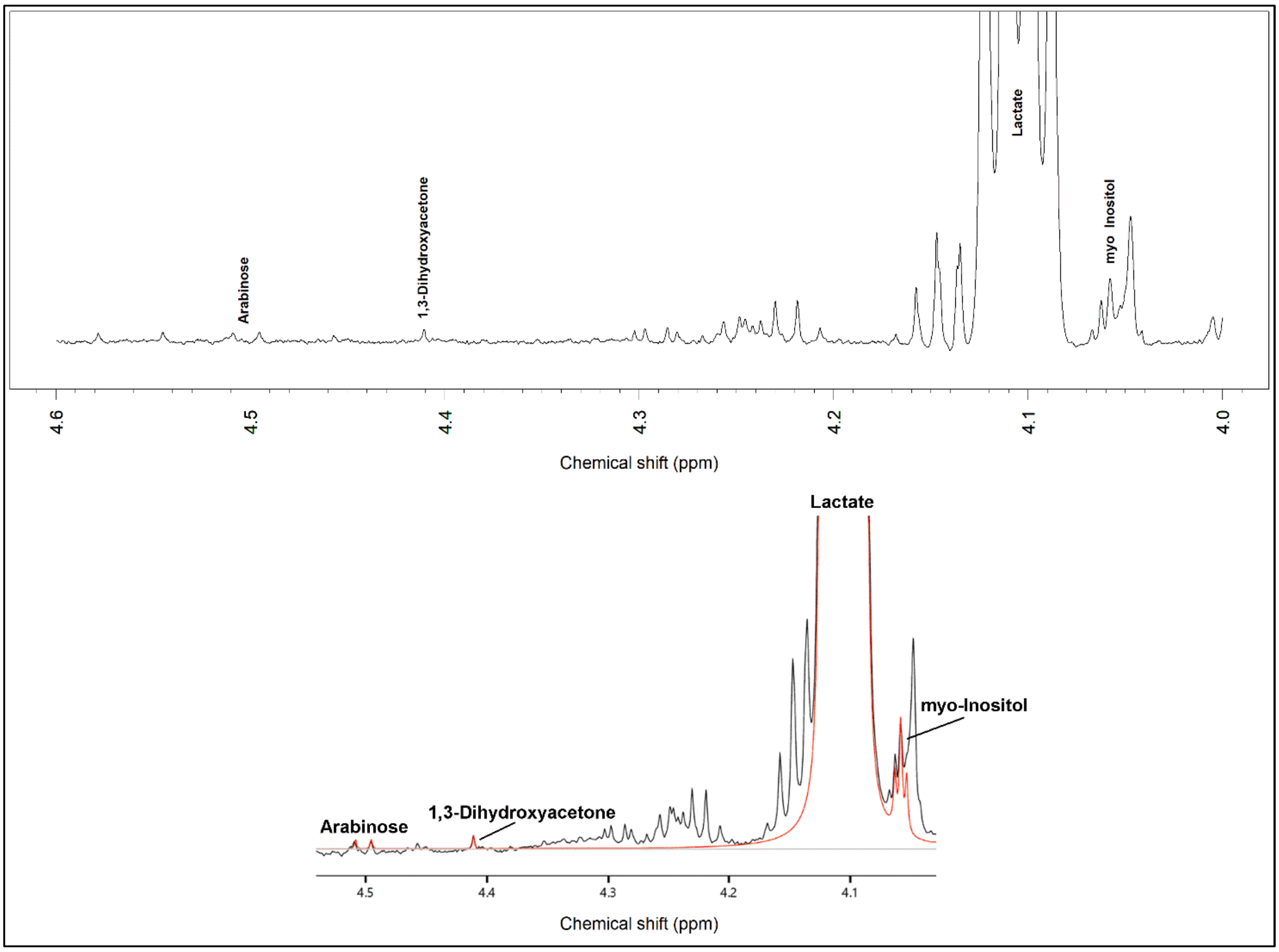
Arabinose
1,3-Dihydroxyacetone
Lactate
myo Inositol
4.6
4.5
4.4
4.3
4.2
4.1
4.0
Chemical shift (ppm)
Lactate
myo-Inositol
Arabinose
1,3-Dihydroxyacetone
4.5
4.4
4.3
4.2
4.1
Chemical shift (ppm)

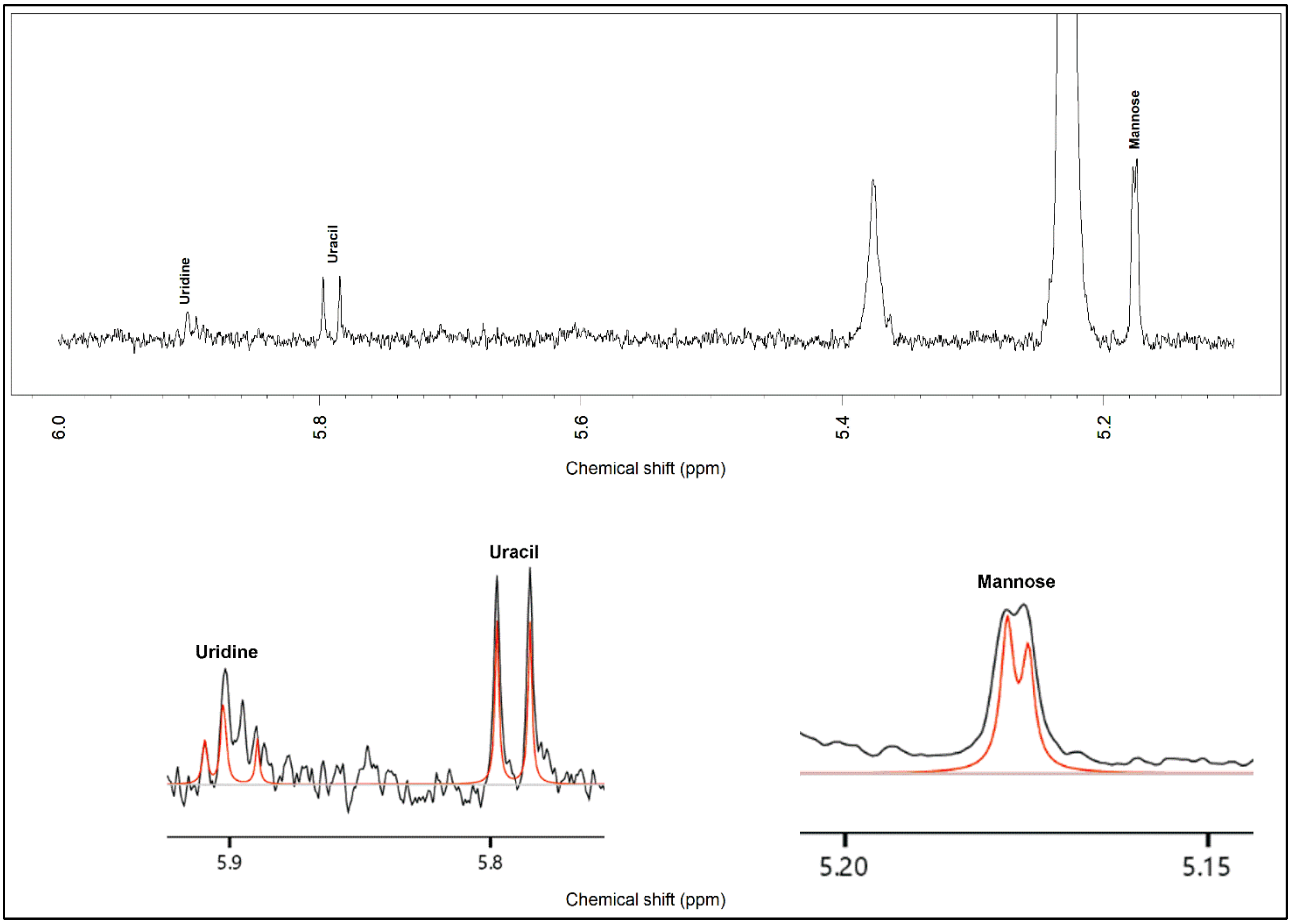
Uridine
Uracil
Mannose
6.0
5.8
5.6
5.4
5.2
Chemical shift (ppm)
Uracil
Uridine
Mannose
5.9
5.8
5.20
5.15
Chemical shift (ppm)

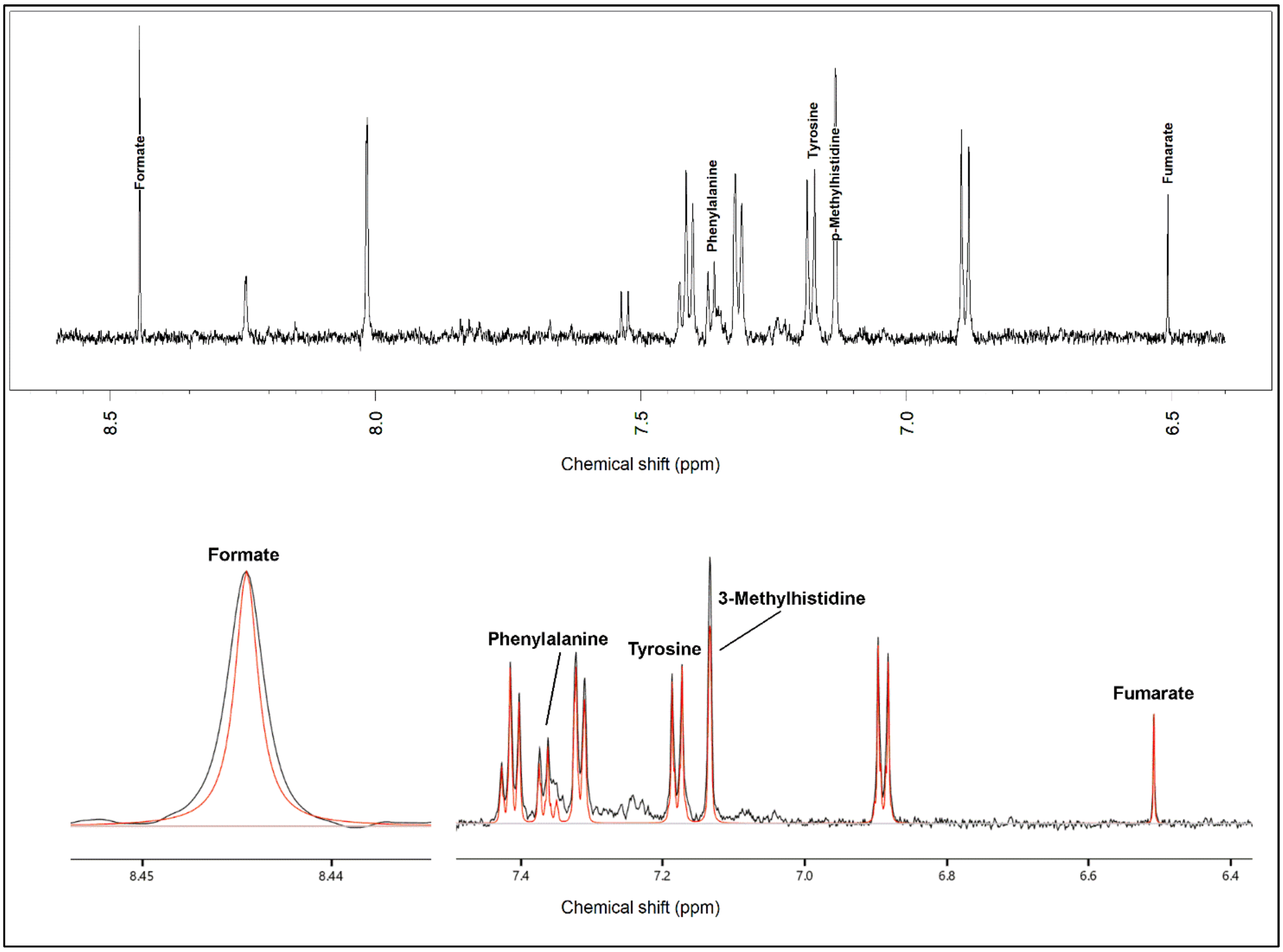
Formate
Phenylalanine
Tyrosine
p-Methylhistidine
Fumarate
8.5
8.0
7.5
7.0
6.5
Chemical shift (ppm)
Formate
Phenylalanine
Tyrosine
3-Methylhistidine
Fumarate
8.45
8.44
7.4
7.2
7.0
6.8
6.6
6.4
Chemical shift (ppm)

Yak Feces

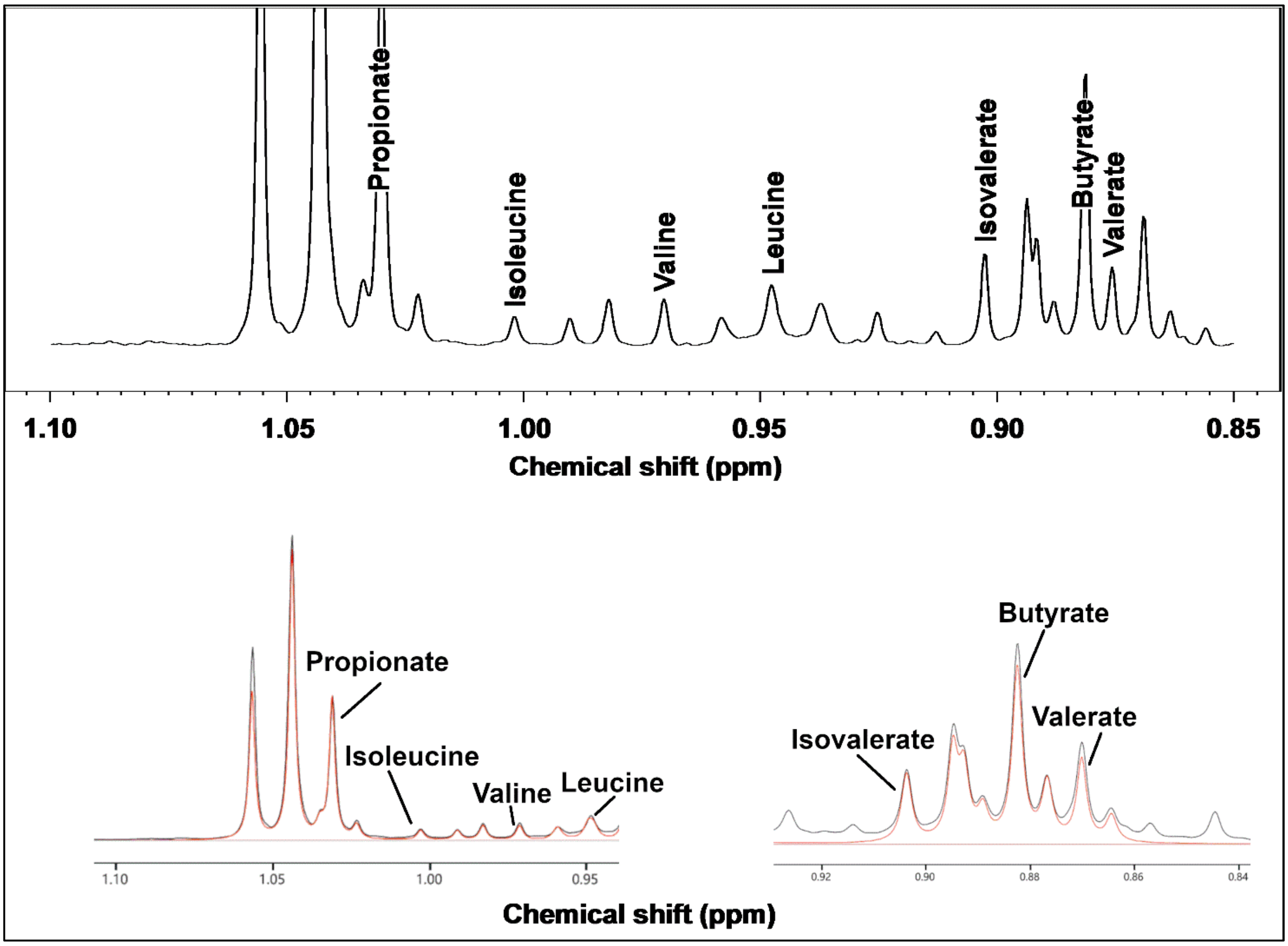
Propionate
Isoleucine
Valine
Leucine
Isovalerate
Butyrate
Valerate
1.10
1.05
1.00
0.95
0.90
0.85
Chemical shift (ppm)
Propionate
Isoleucine
Valine
Leucine
Butyrate
Isovalerate
Valerate
Chemical shift (ppm)

11

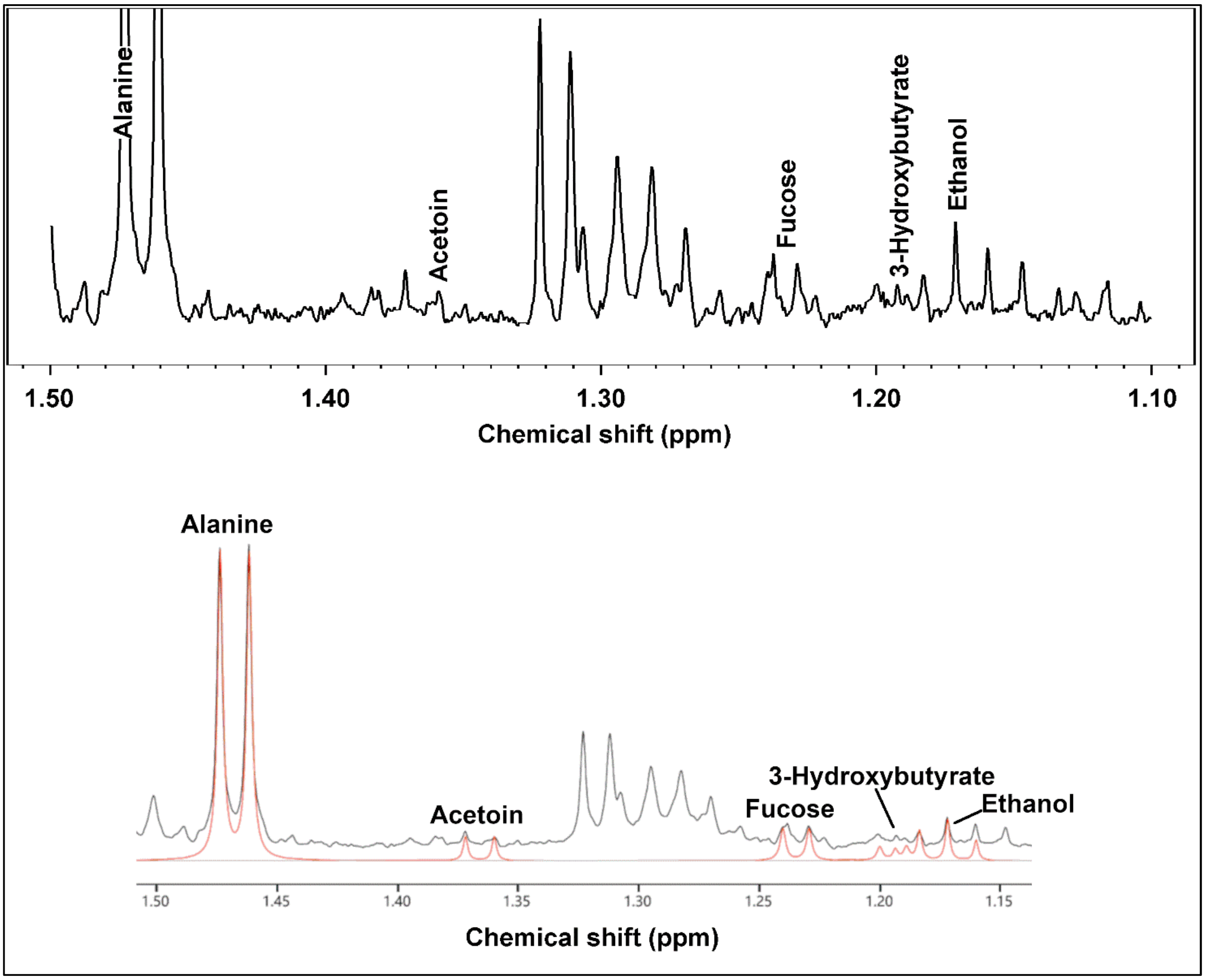

Alanine
Acetoin
Fucose
3-Hydroxybutyrate
Ethanol
1.50
1.40
1.30
1.20
1.10
Chemical shift (ppm)
Alanine
Acetoin
Fucose
3-Hydroxybutyrate
Ethanol
1.50
1.45
1.40
1.35
1.30
1.25
1.20
1.15
Chemical shift (ppm)

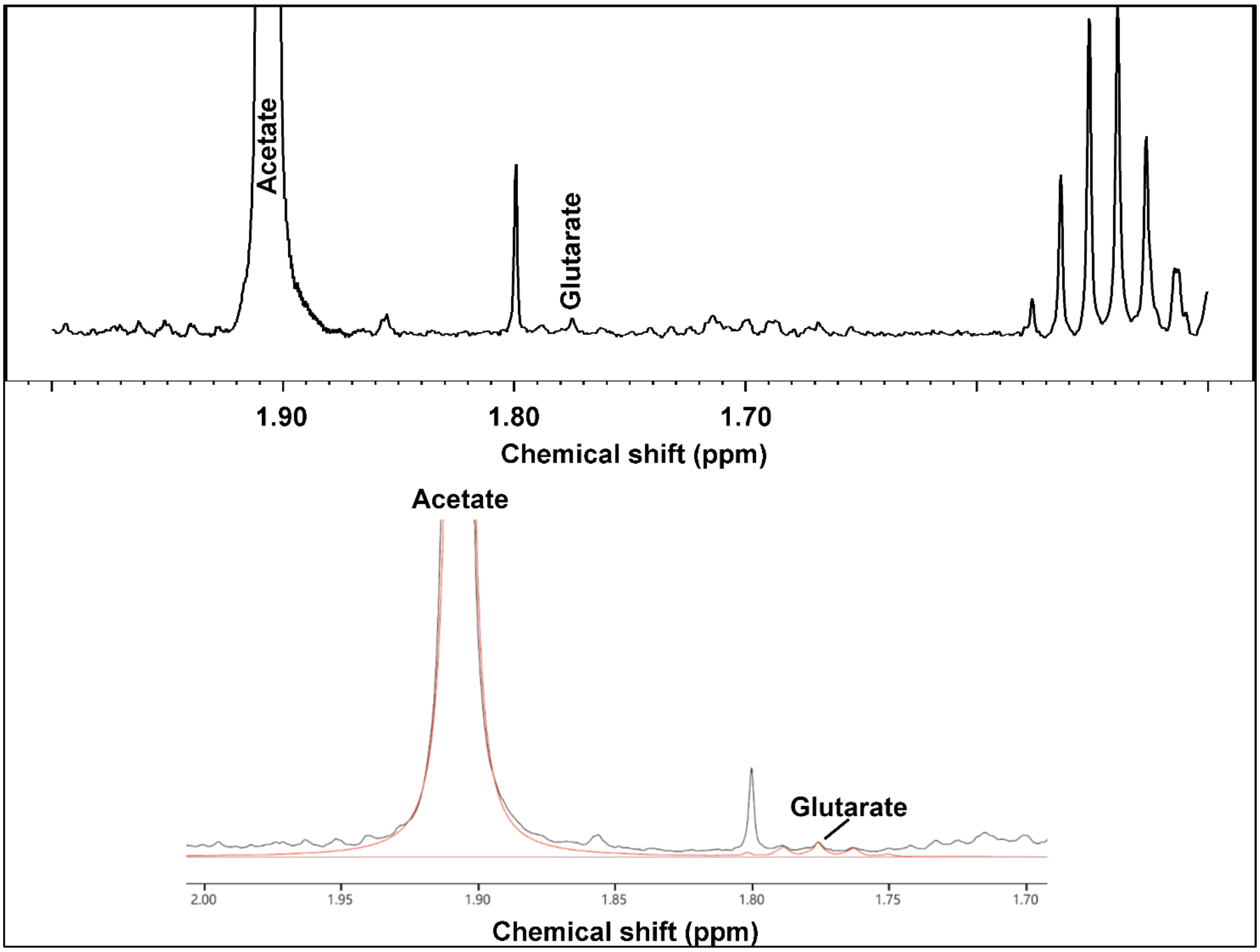
Acetate
Glutarate
1.90
1.80
1.70
Chemical shift (ppm)
Acetate
Glutarate
2.00
1.95
1.90
1.85
1.80
1.75
1.70
Chemical shift (ppm)

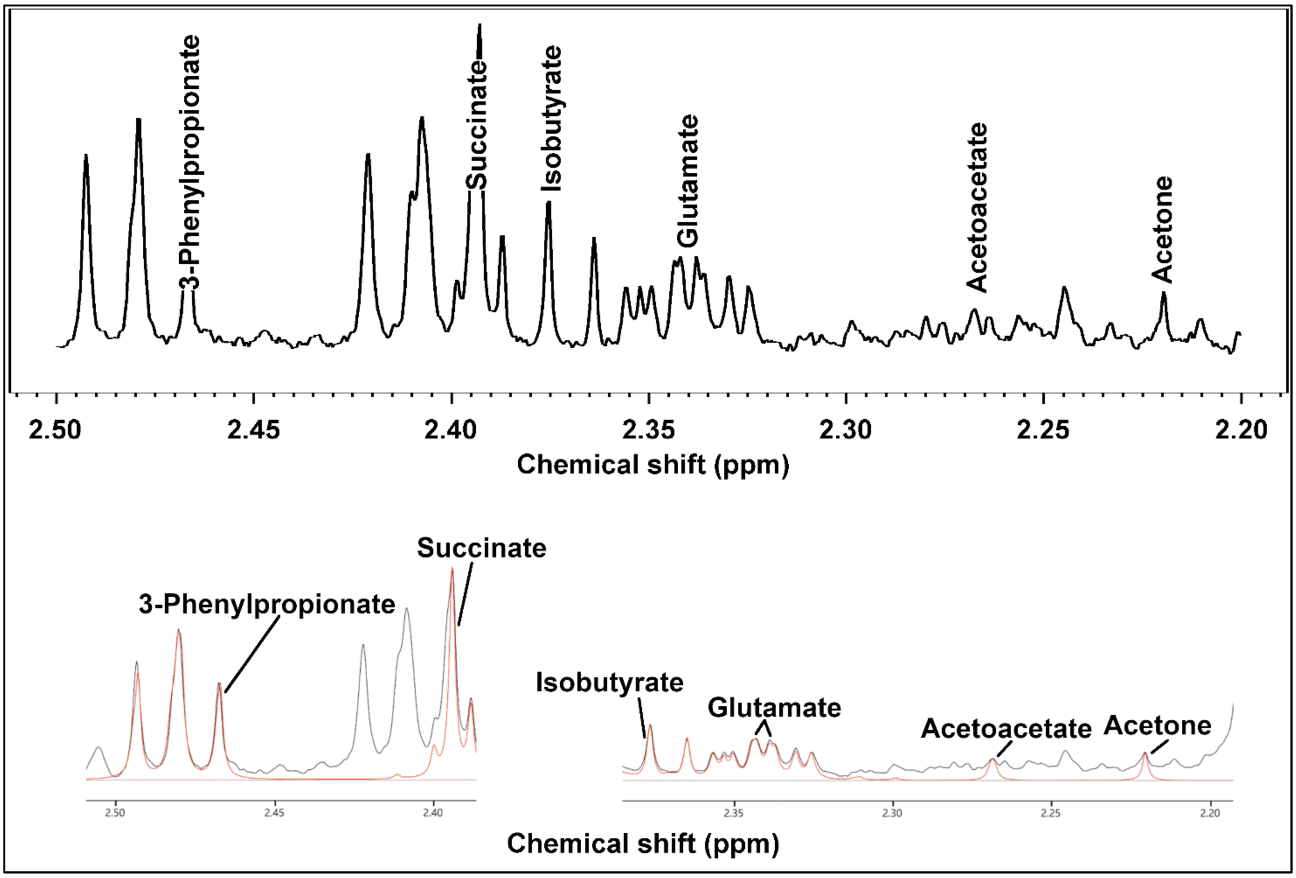
3-Phenylpropionate
Succinate
Isobutyrate
Glutamate
Acetoacetate
Acetone
2.50
2.45
2.40
2.35
2.30
2.25
2.20
Chemical shift (ppm)
Succinate
3-Phenylpropionate
Isobutyrate
Glutamate
Acetoacetate
Acetone
Chemical shift (ppm)

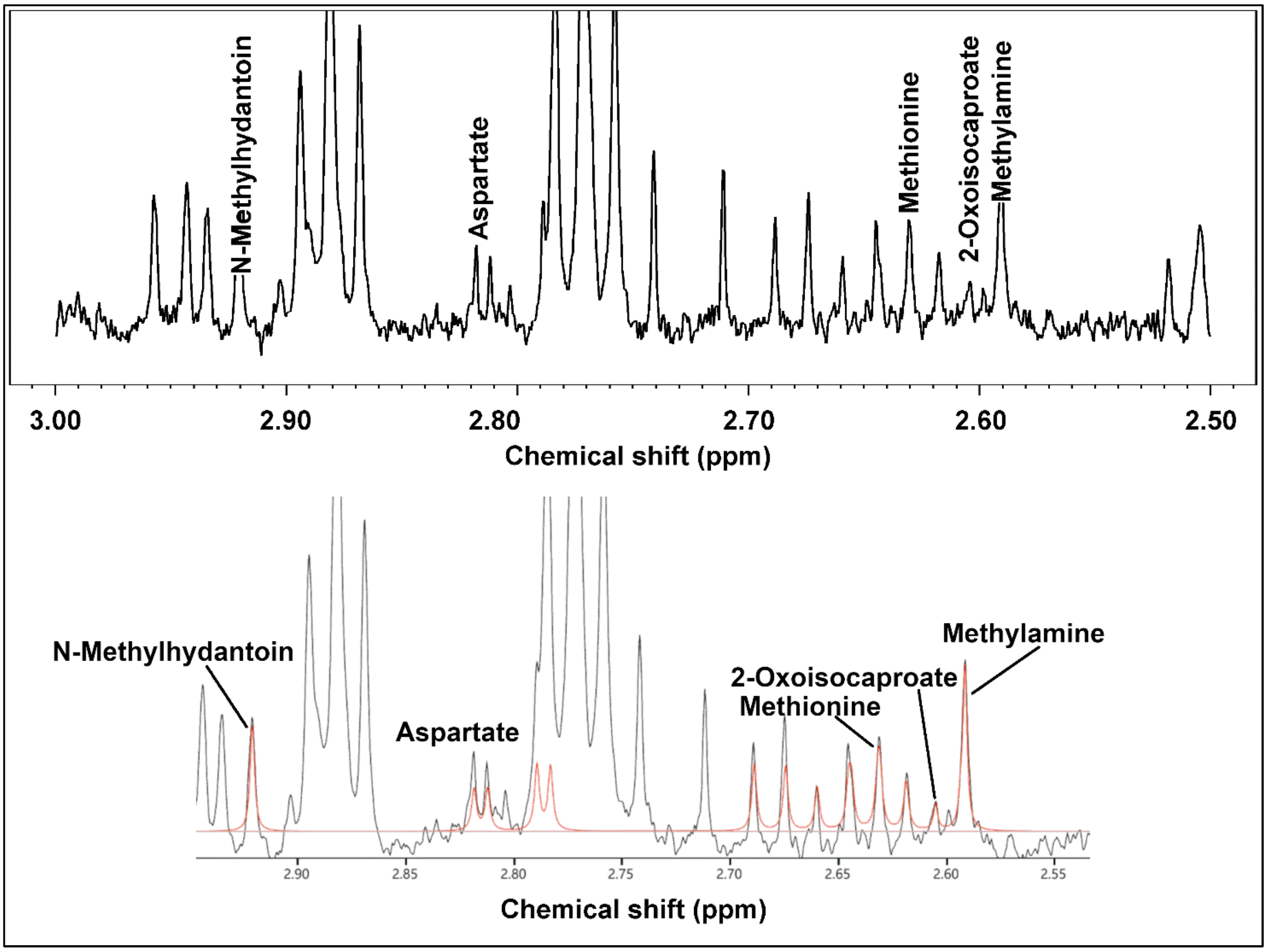
N-Methylhydantoin
Aspartate
Methionine
2-Oxoisocaproate
Methylamine
3.00
2.90
2.80
2.70
2.60
2.50
Chemical shift (ppm)
N-Methylhydantoin
Aspartate
2-Oxoisocaproate
Methionine
Methylamine
2.90
2.85
2.80
2.75
2.70
2.65
2.60
2.55
Chemical shift (ppm)

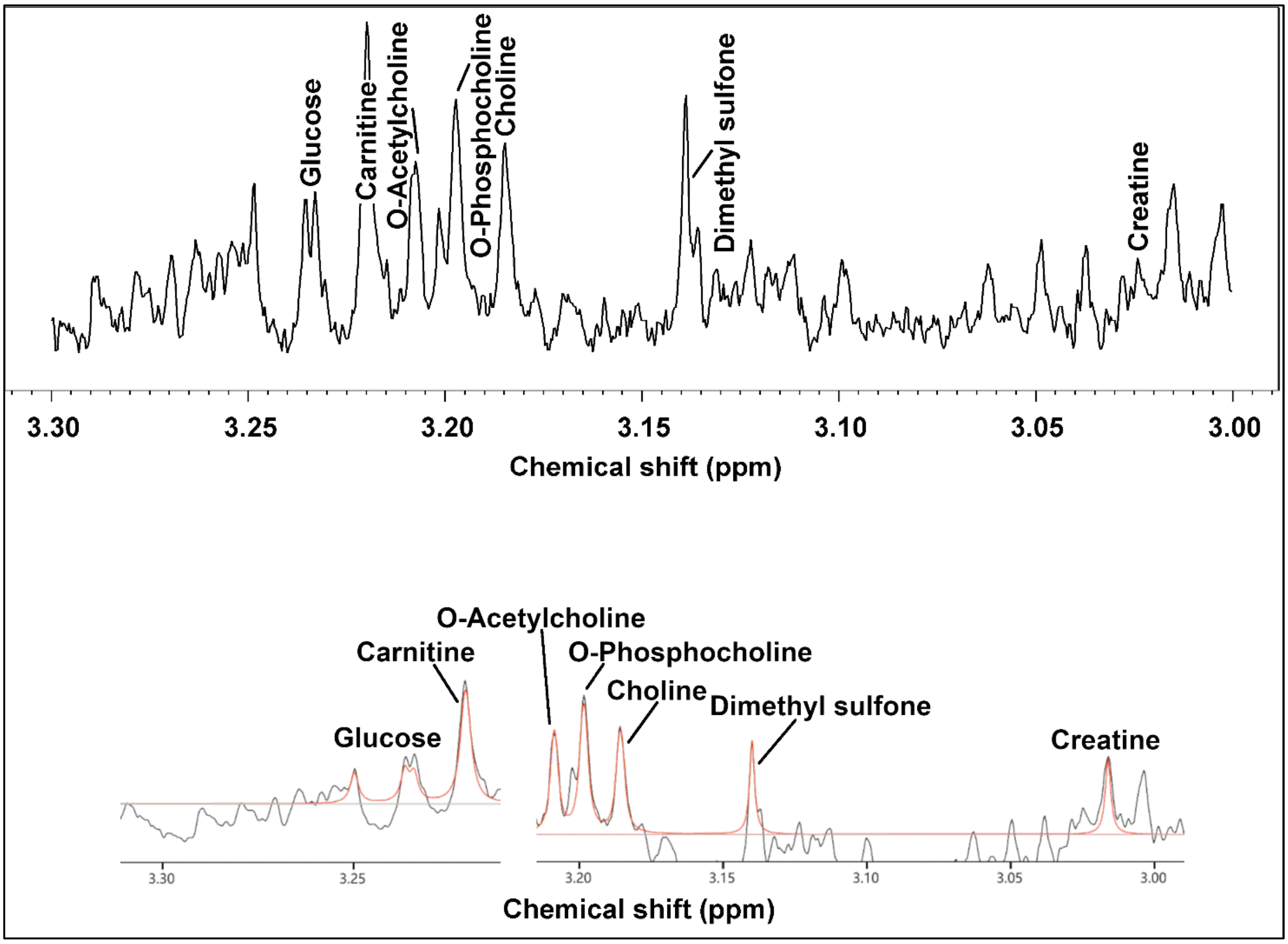

Glucose
Carnitine
O-Acetylcholine
O-Phosphocholine
Choline
Dimethyl sulfone
Creatine
3.30
3.25
3.20
3.15
3.10
3.05
3.00
Chemical shift (ppm)
O-Acetylcholine
Carnitine
O-Phosphocholine
Choline
Dimethyl sulfone
Glucose
Creatine
Chemical shift (ppm)

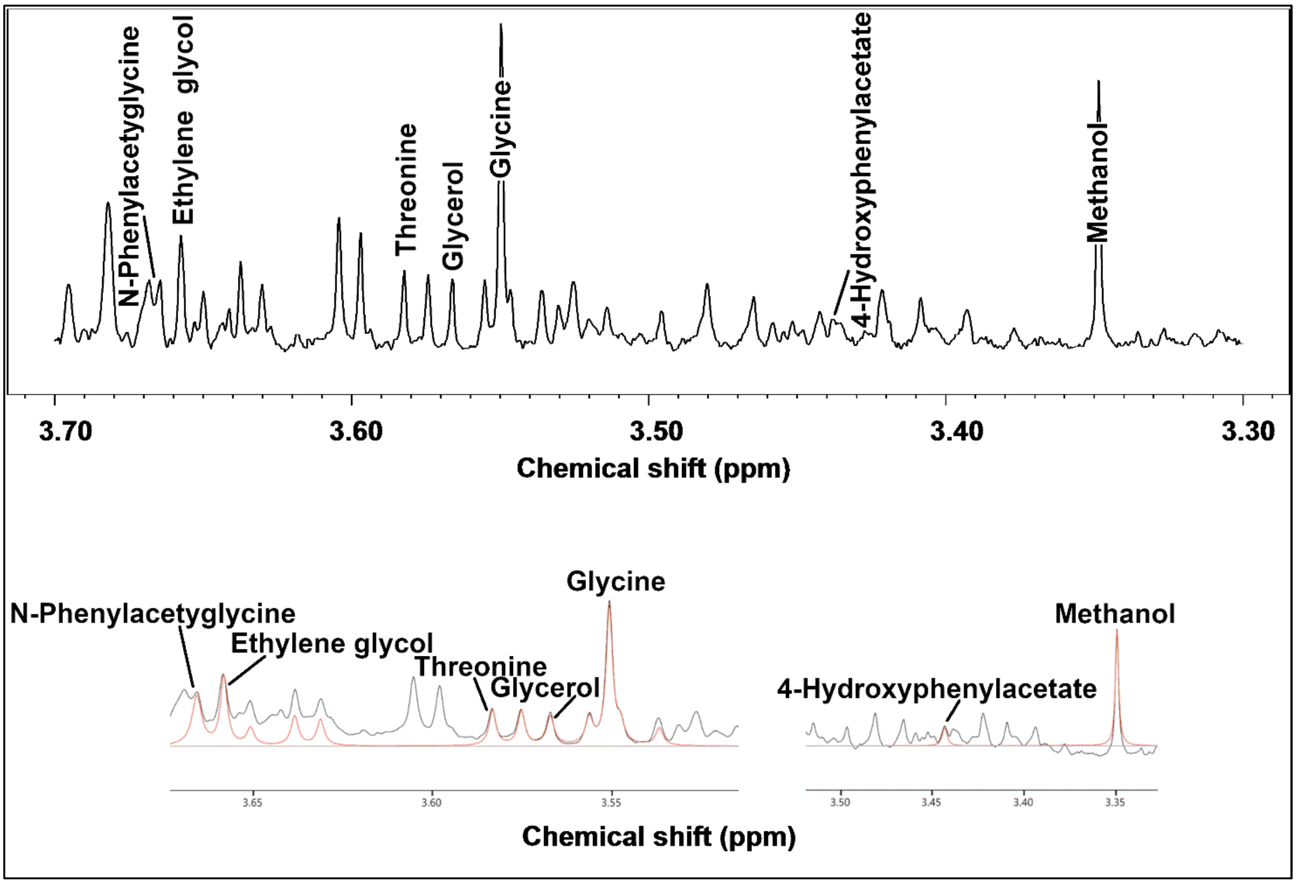

N-Phenylacetyglycine
Ethylene glycol
Threonine
Glycerol
Glycine
4-Hydroxyphenylacetate
Methanol
3.70
3.60
3.50
3.40
3.30
Chemical shift (ppm)
Glycine
N-Phenylacetyglycine
Ethylene glycol
Threonine
Glycerol
Methanol
4-Hydroxyphenylacetate
Chemical shift (ppm)

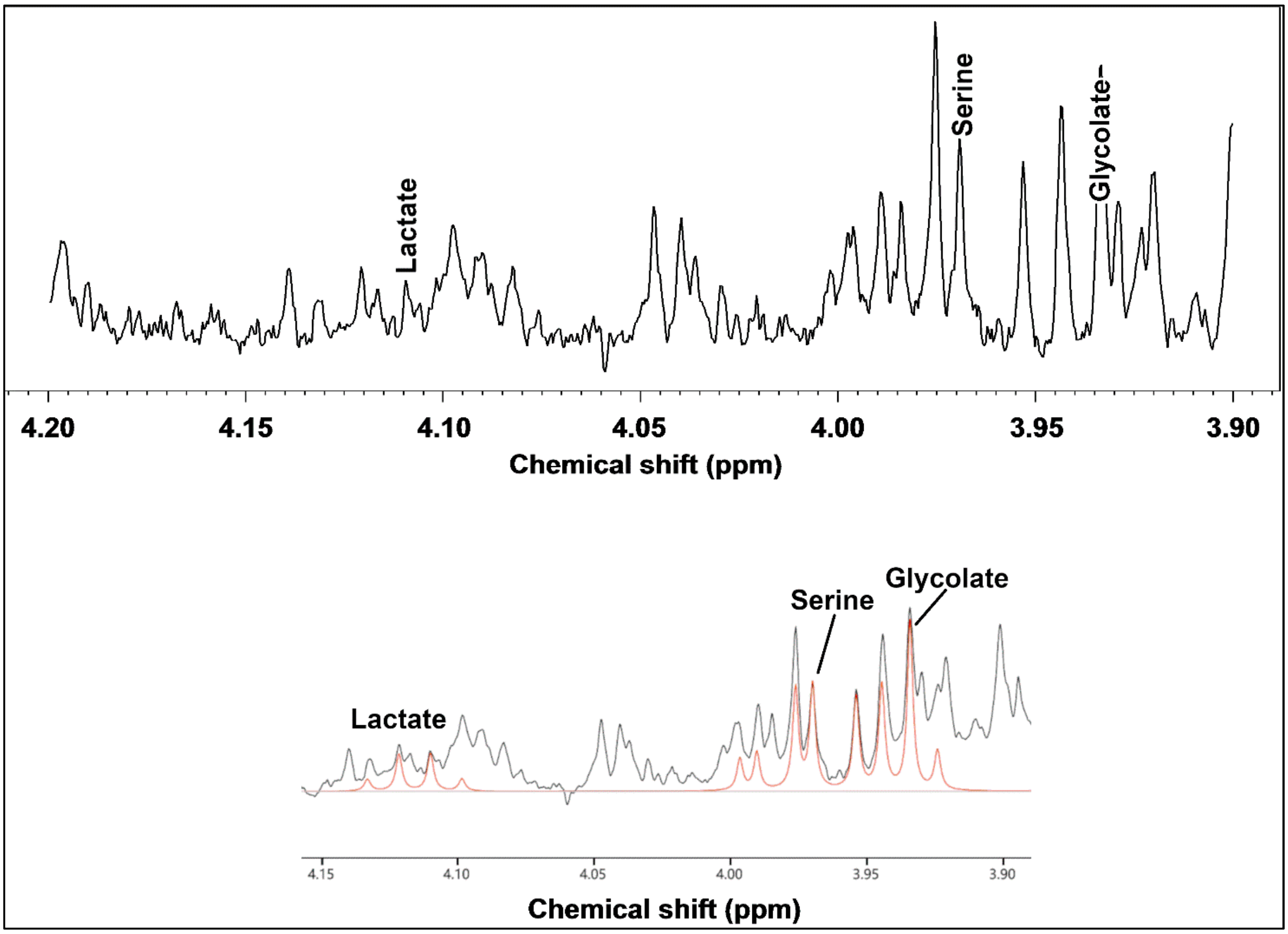

Lactate
Serine
Glycolate
4.20
4.15
4.10
4.05
4.00
3.95
3.90
Chemical shift (ppm)
Lactate
Serine
Glycolate
4.15
4.10
4.05
4.00
3.95
3.90
Chemical shift (ppm)

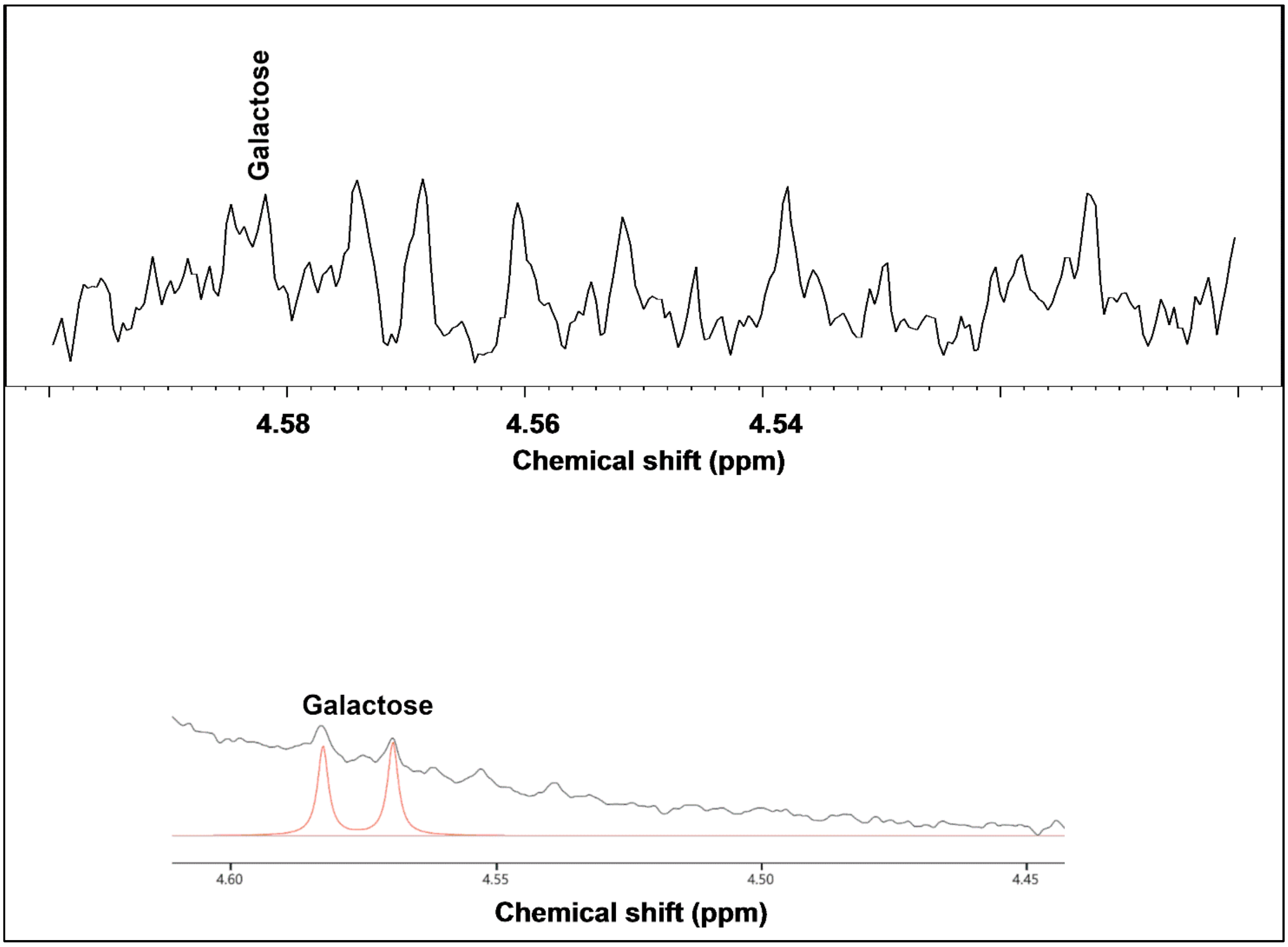
Galactose
4.58
4.56
4.54
Chemical shift (ppm)
Galactose
4.60
4.55
4.50
4.45
Chemical shift (ppm)

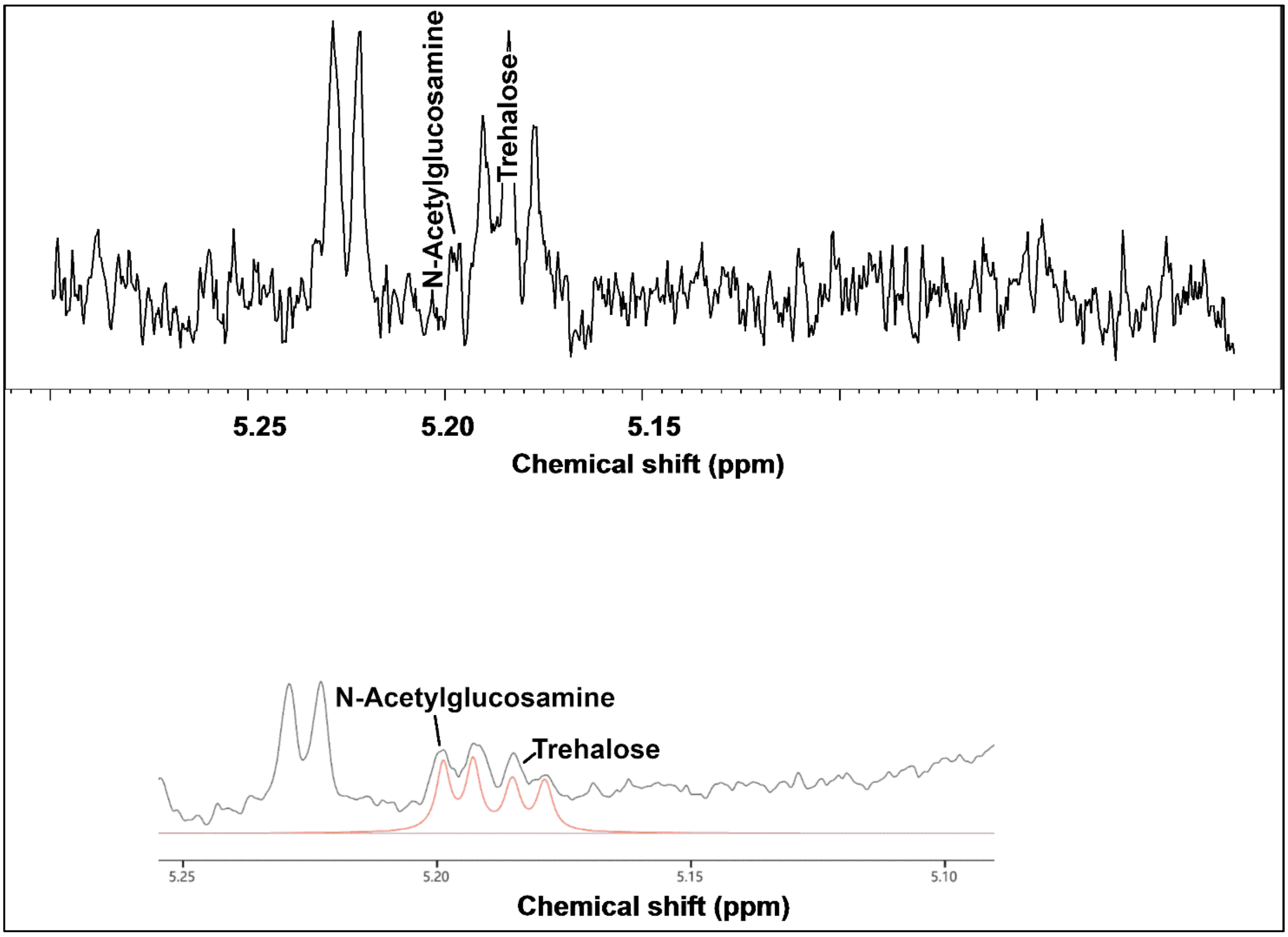

N-Acetylglucosamine
Trehalose
5.25
5.20
5.15
Chemical shift (ppm)
N-Acetylglucosamine
Trehalose
5.25
5.20
5.15
5.10
Chemical shift (ppm)

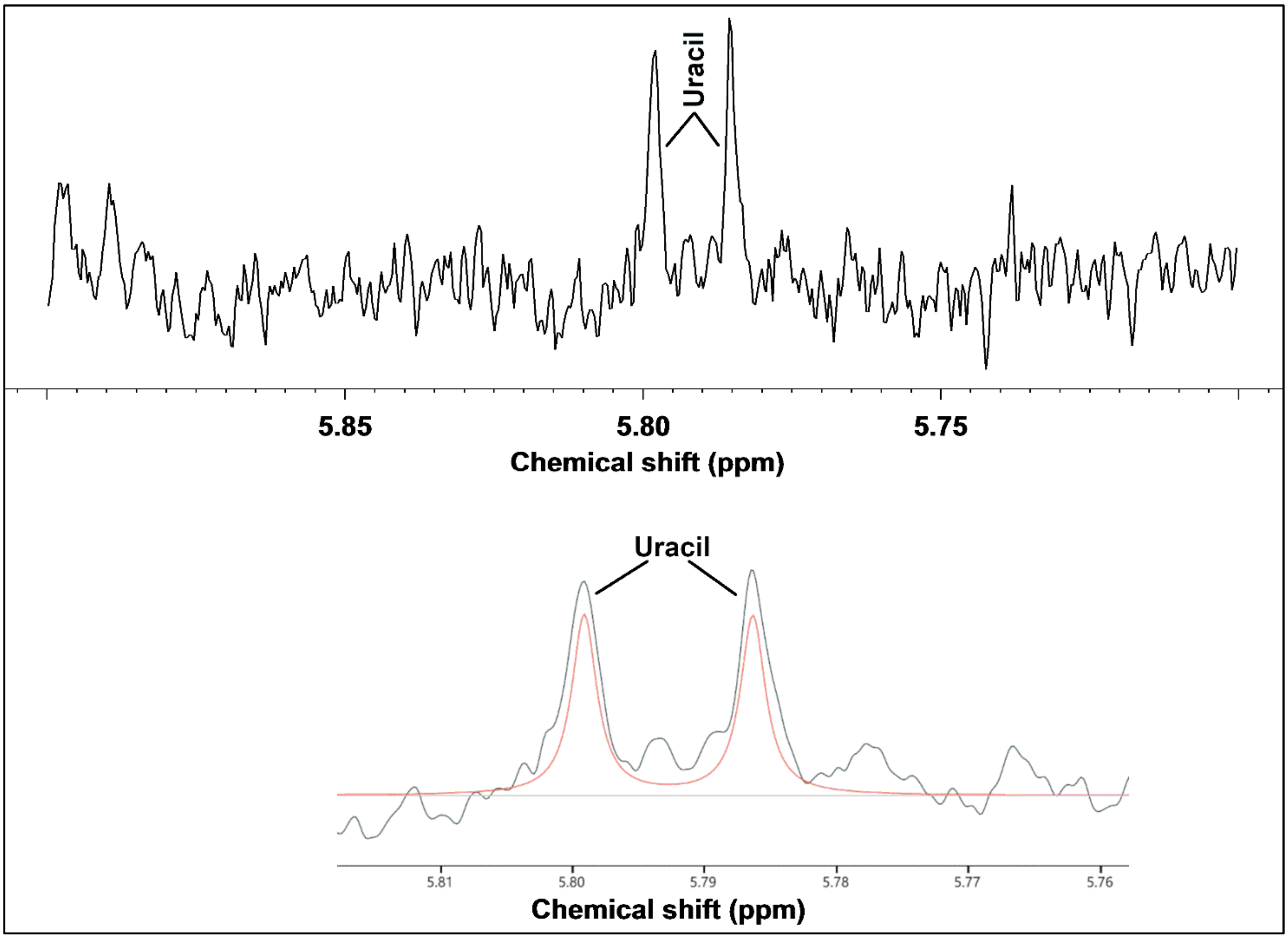
Uracil
5.85
5.80
5.75
Chemical shift (ppm)
Uracil
5.81
5.80
5.79
5.78
5.77
5.76
Chemical shift (ppm)

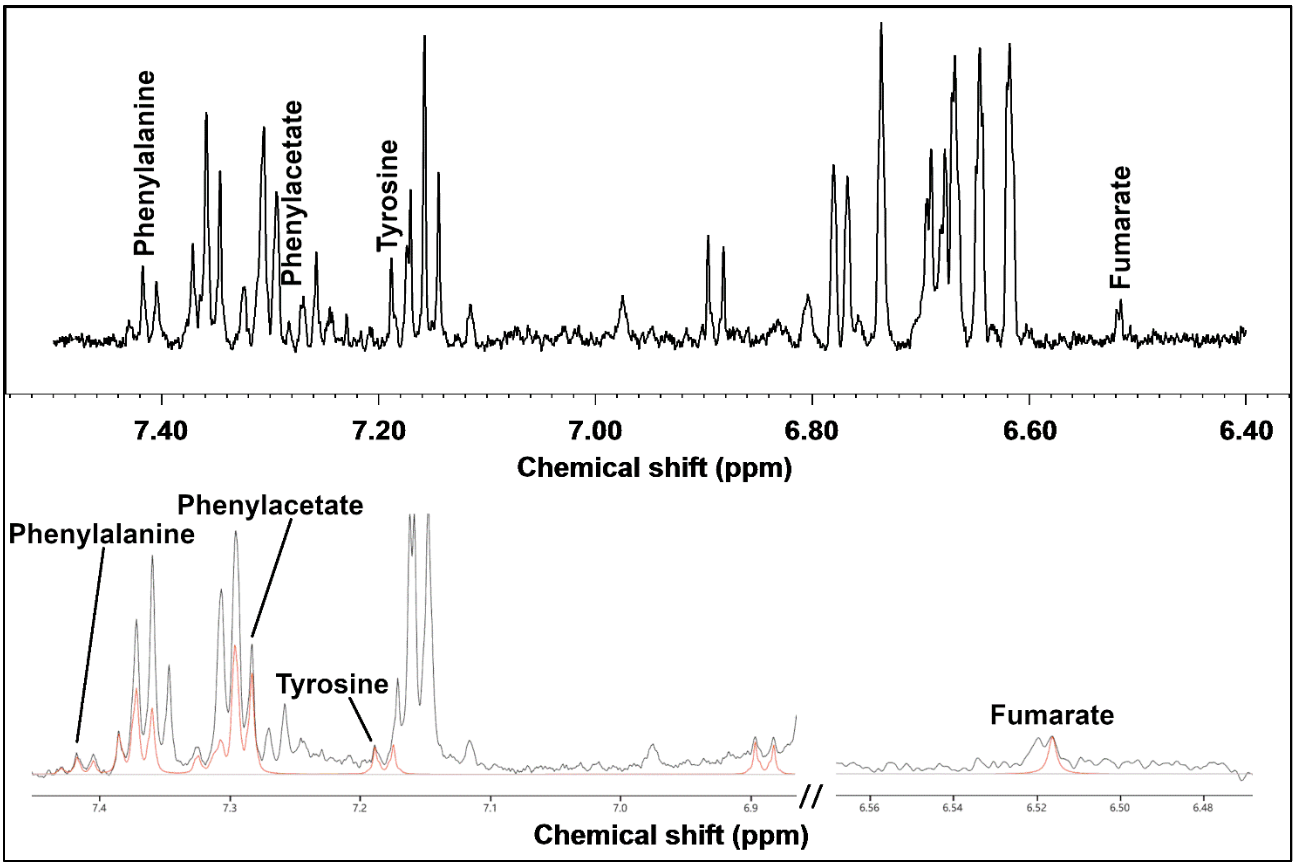
Phenylalanine
Phenylacetate
Tyrosine
Fumarate
7.40
7.20
7.00
6.80
6.60
6.40
Chemical shift (ppm)
Phenylacetate
Phenylalanine
Tyrosine
Fumarate
Chemical shift (ppm)

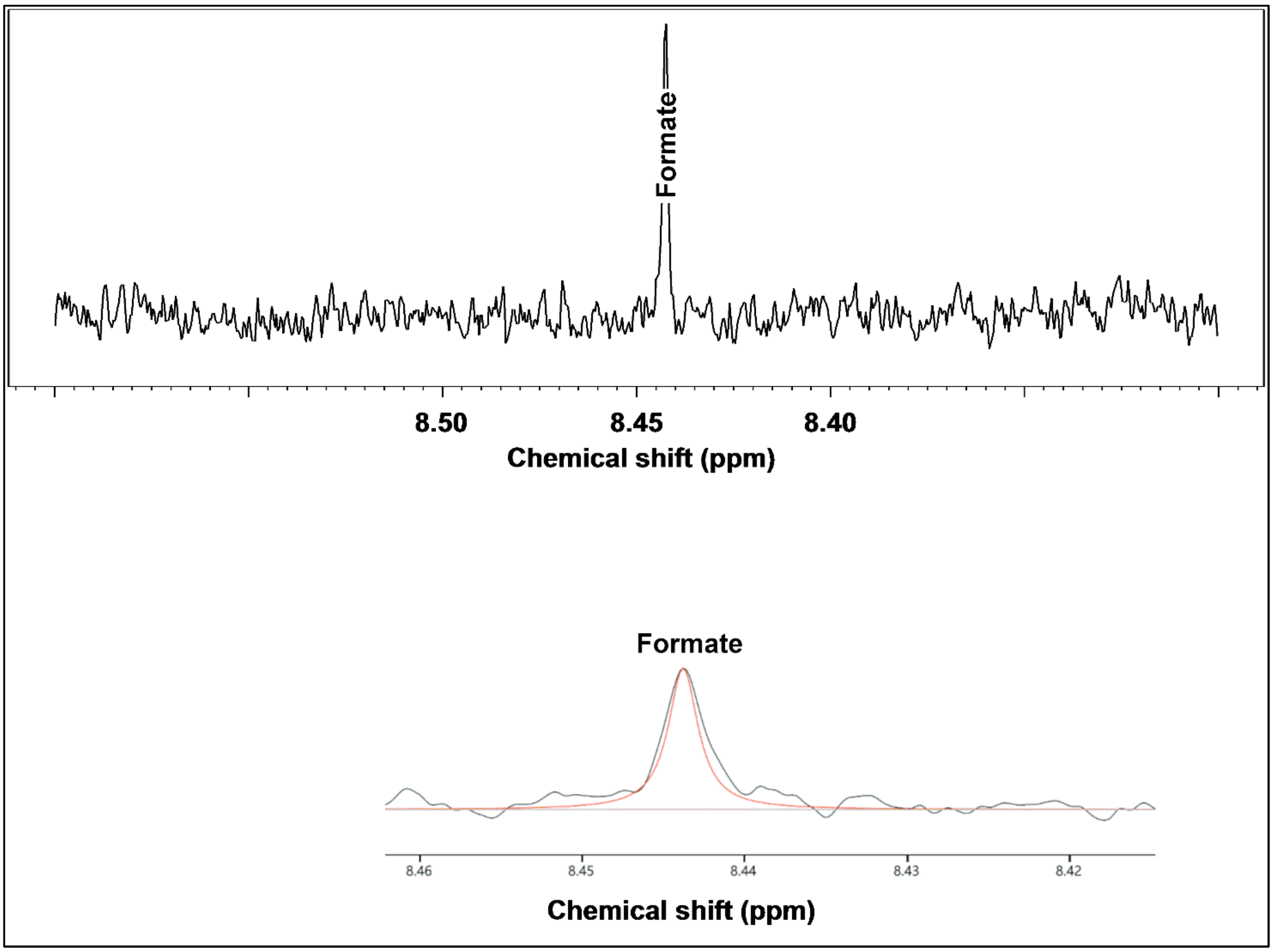
Formate
8.50
8.45
8.40
Chemical shift (ppm)
Formate
8.46
8.45
8.44
8.43
8.42
Chemical shift (ppm)

Yak Urine

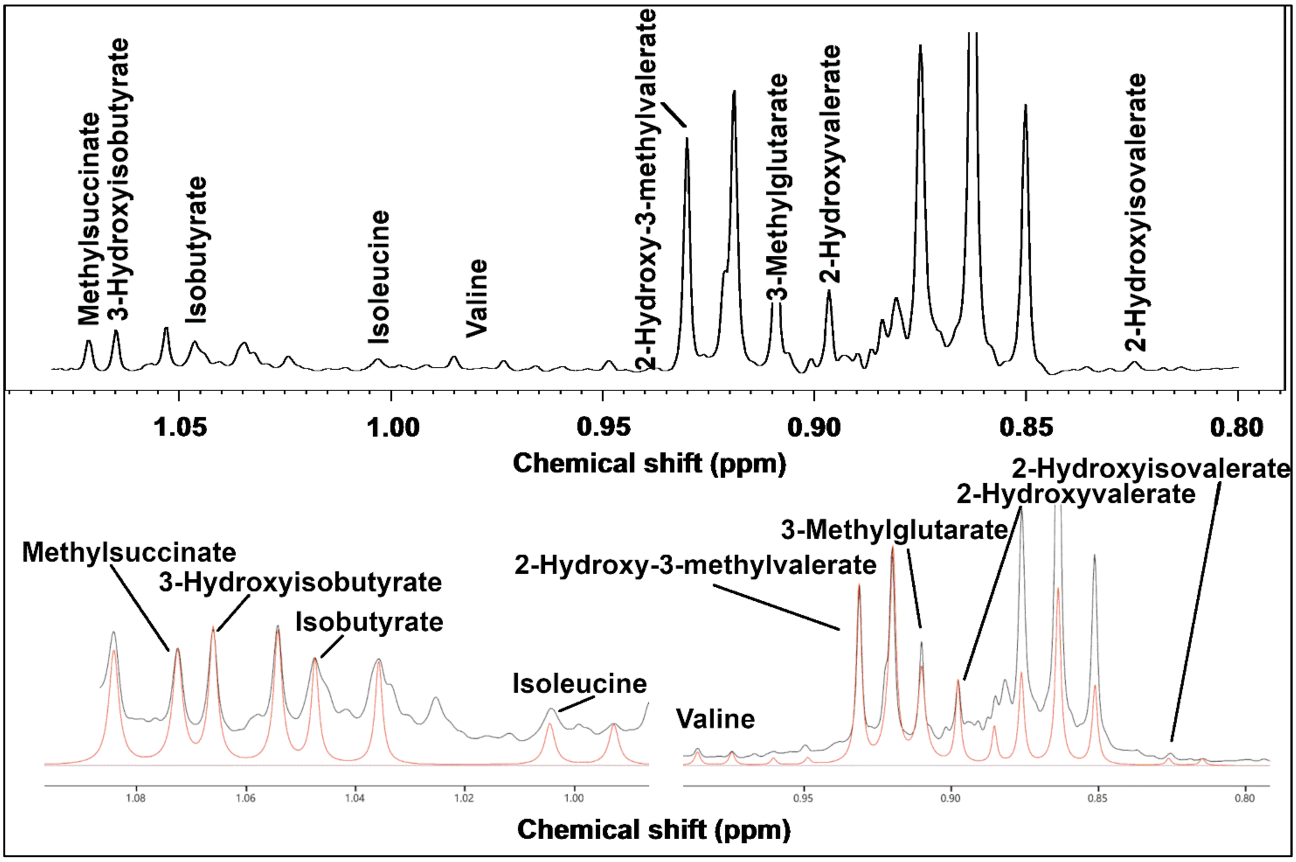
Methylsuccinate
3-Hydroxyisobutyrate
Isobutyrate
Isoleucine
Valine
2-Hydroxy-3-methylvalerate
3-Methylglutarate
2-Hydroxyvalerate
2-Hydroxyisovalerate
1.05
1.00
0.95
0.90
0.85
0.80
Chemical shift (ppm)
1.08
1.06
1.04
1.02
1.00
0.95
0.90
0.85
0.80
Chemical shift (ppm)

24

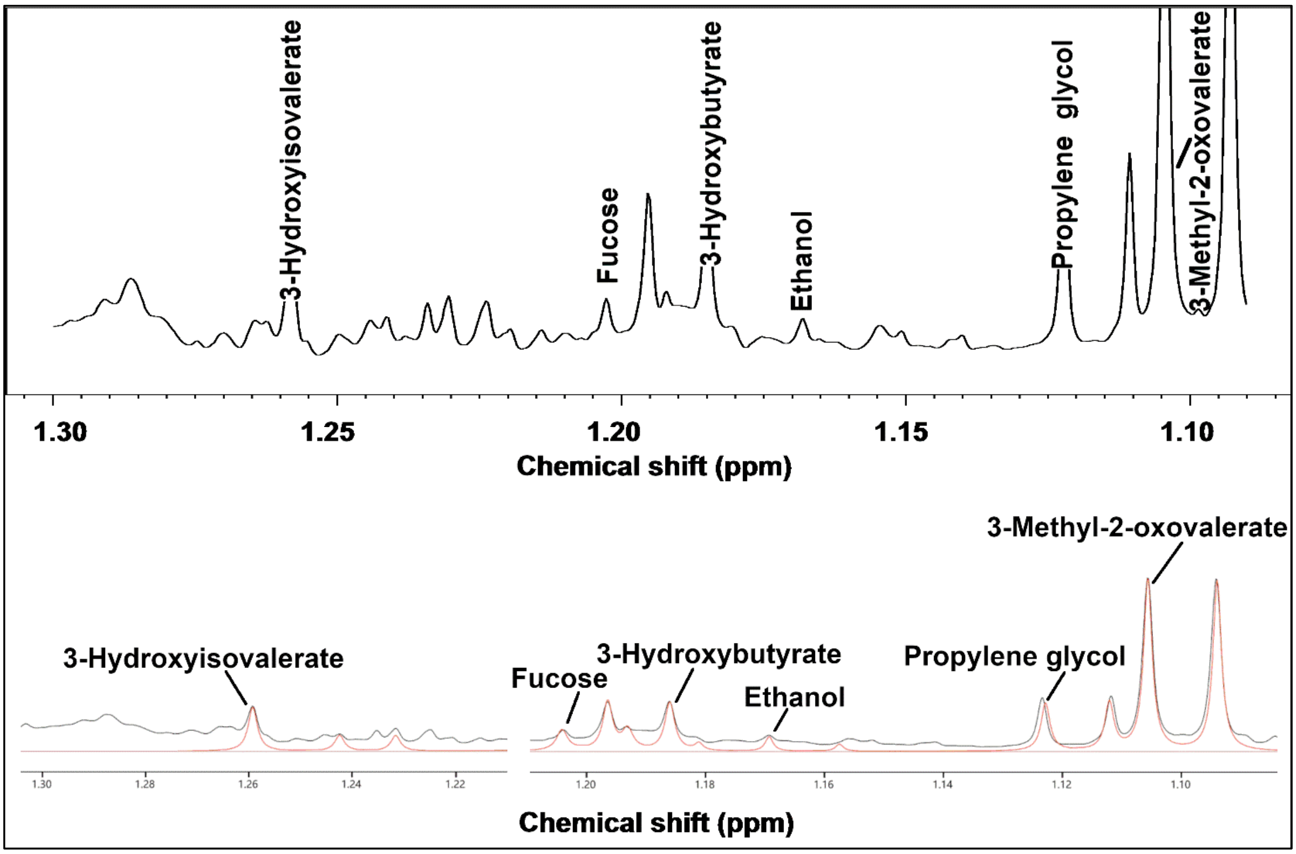
3-Hydroxyisovalerate
Fucose
3-Hydroxybutyrate
Ethanol
Propylene glycol
3-Methyl-2-oxovalerate
1.30
1.25
1.20
1.15
1.10
Chemical shift (ppm)
1.30
1.28
1.26
1.24
1.22
1.20
1.18
1.16
1.14
1.12
1.10
Chemical shift (ppm)

25

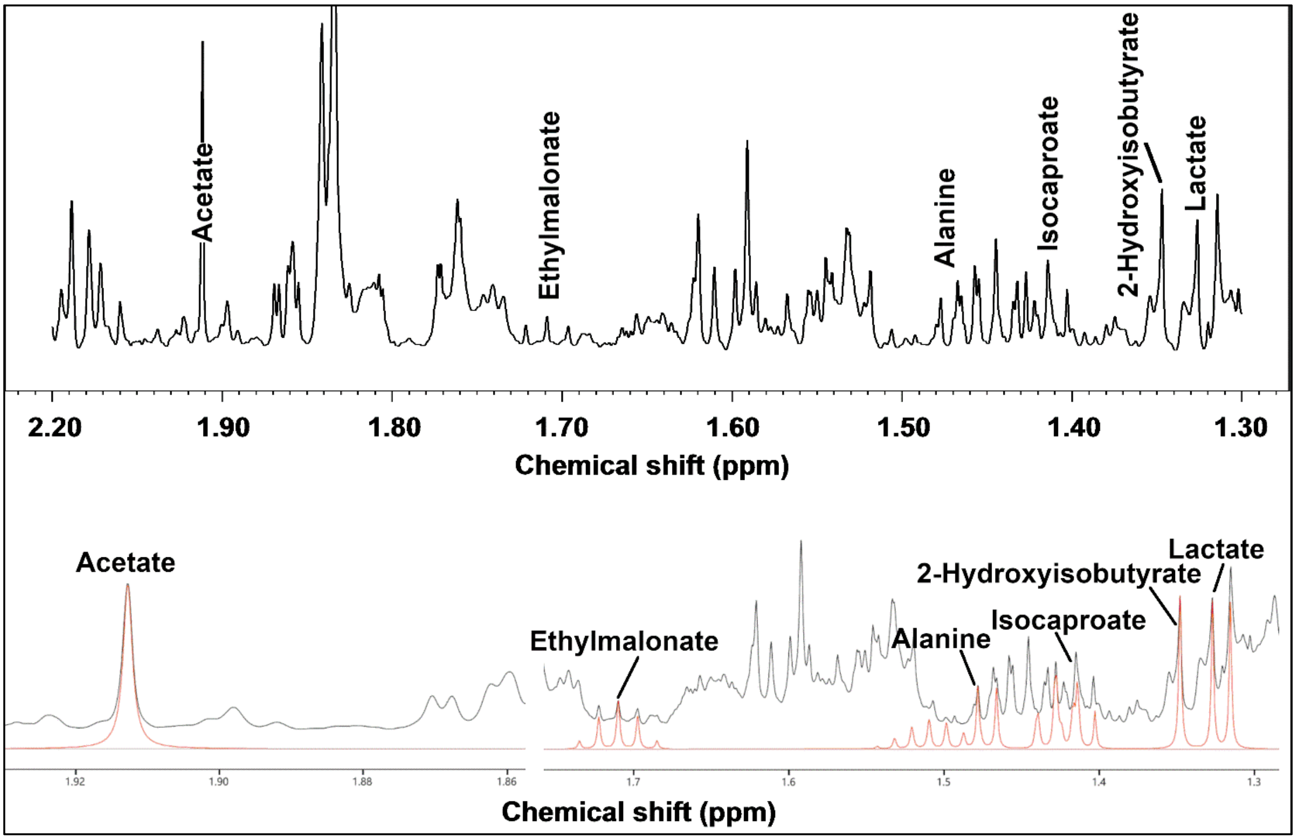
Acetate
Ethylmalonate
Alanine
Isocaproate
2-Hydroxyisobutyrate
Lactate
2.20
1.90
1.80
1.70
1.60
1.50
1.40
1.30
Chemical shift (ppm)
Chemical shift (ppm)

26

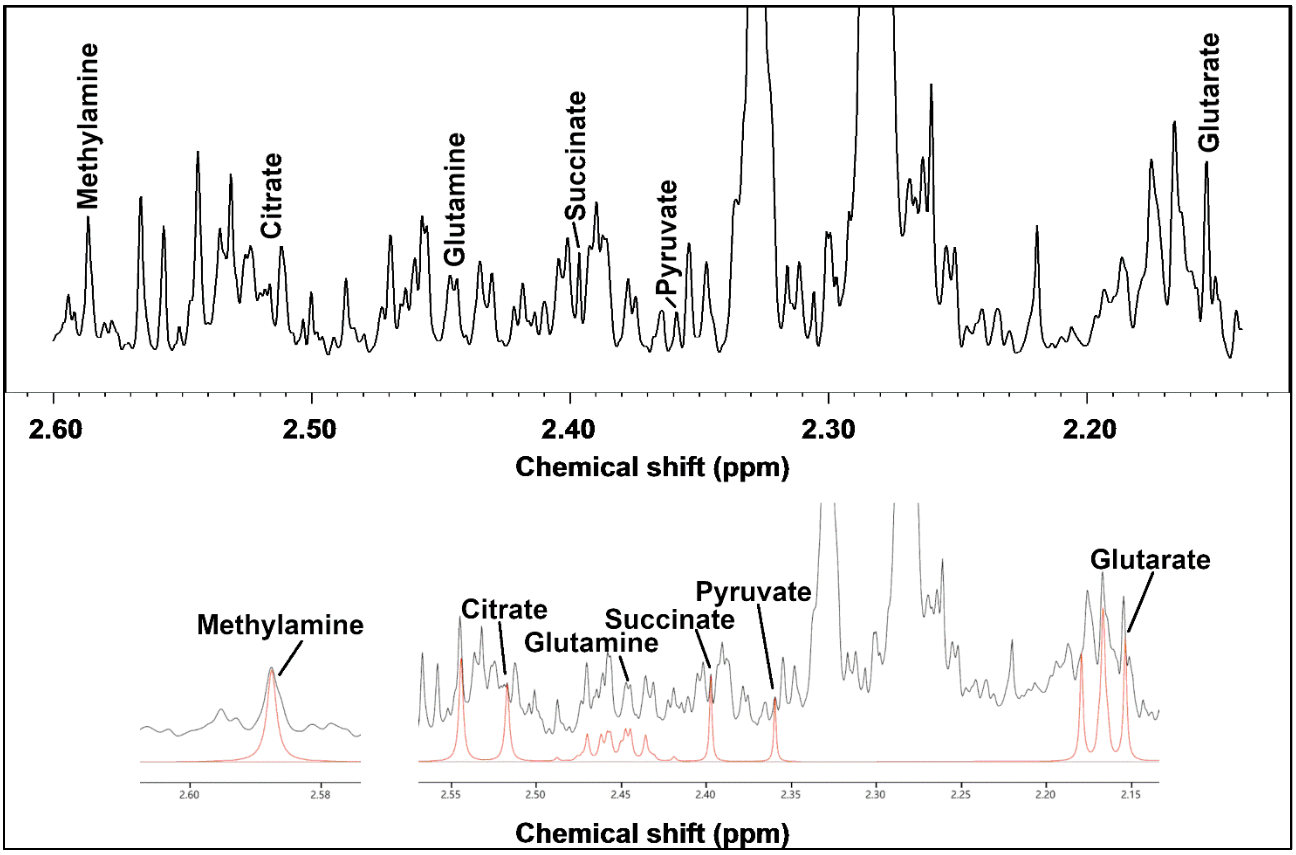
Methylamine
Citrate
Glutamine
Succinate
Pyruvate
Glutarate
2.60
2.50
2.40
2.30
2.20
Chemical shift (ppm)
Chemical shift (ppm)

27

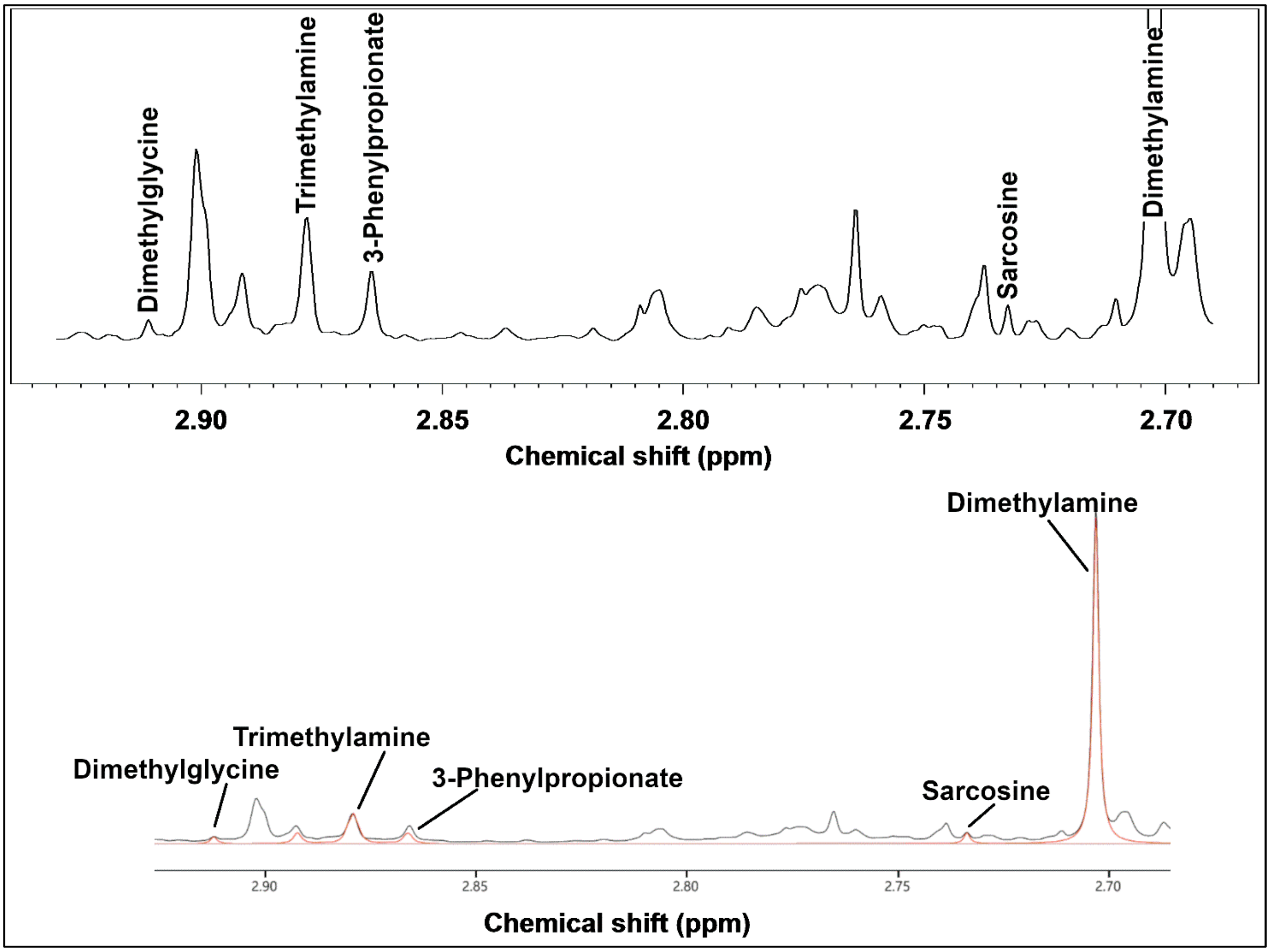
Dimethylglycine
Trimethylamine
3-Phenylpropionate
Sarcosine
Dimethylamine
2.90
2.85
2.80
2.75
2.70
Chemical shift (ppm)
Dimethylamine
Trimethylamine
Dimethylglycine
3-Phenylpropionate
Sarcosine
Chemical shift (ppm)

28

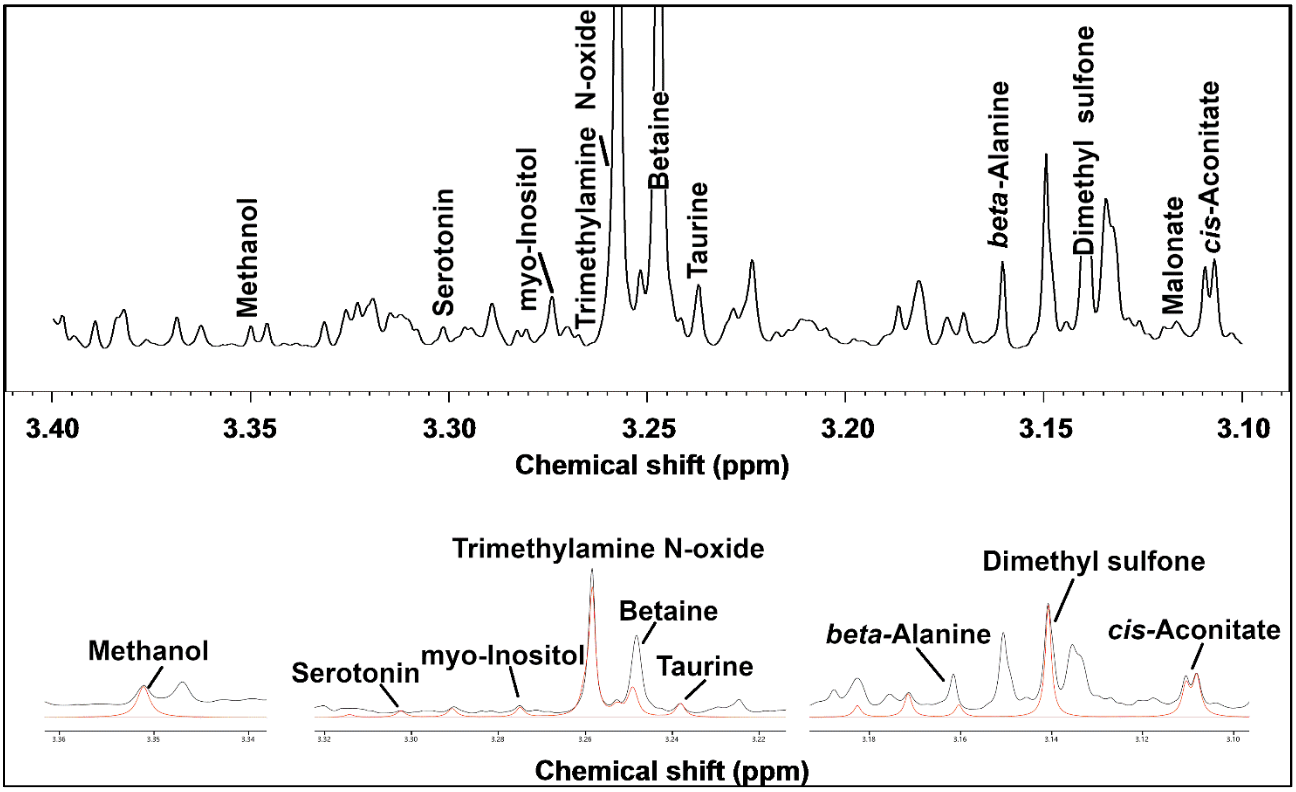
Methanol
Serotonin
myo-Inositol
Trimethylamine N-oxide
Betaine
Taurine
beta-Alanine
Dimethyl sulfone
Malonate
cis-Aconitate
3.40
3.35
3.30
3.25
3.20
3.15
3.10
Chemical shift (ppm)
Trimethylamine N-oxide
Dimethyl sulfone
Betaine
beta-Alanine
cis-Aconitate
Methanol
Taurine
Serotonin
myo-Inositol
Chemical shift (ppm)

29

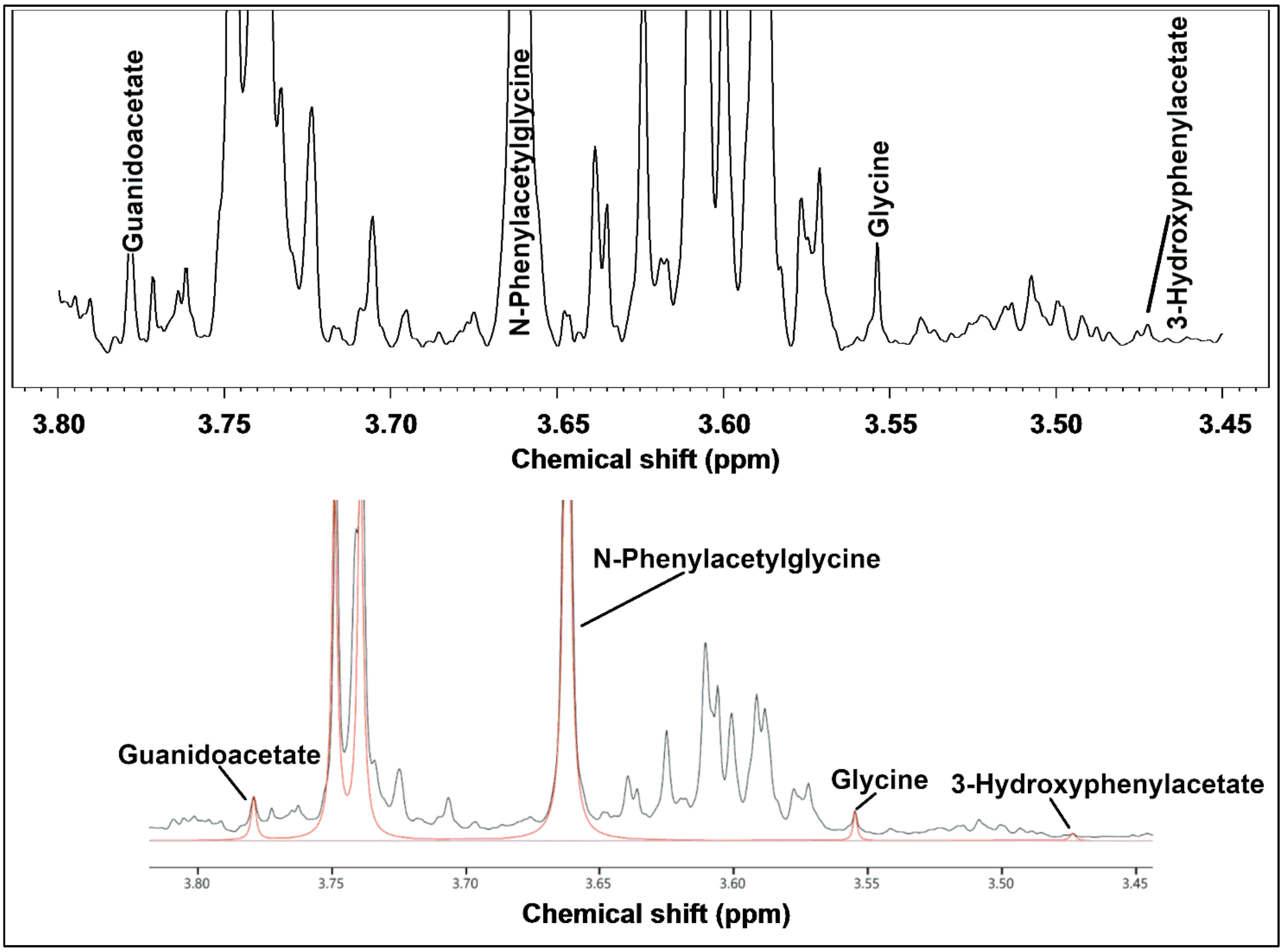
Guanidoacetate
N-Phenylacetylglycine
Glycine
3-Hydroxyphenylacetate
3.80
3.75
3.70
3.65
3.60
3.55
3.50
3.45
Chemical shift (ppm)
N-Phenylacetylglycine
Guanidoacetate
Glycine
3-Hydroxyphenylacetate
3.80
3.75
3.70
3.65
3.60
3.55
3.50
3.45
Chemical shift (ppm)

30

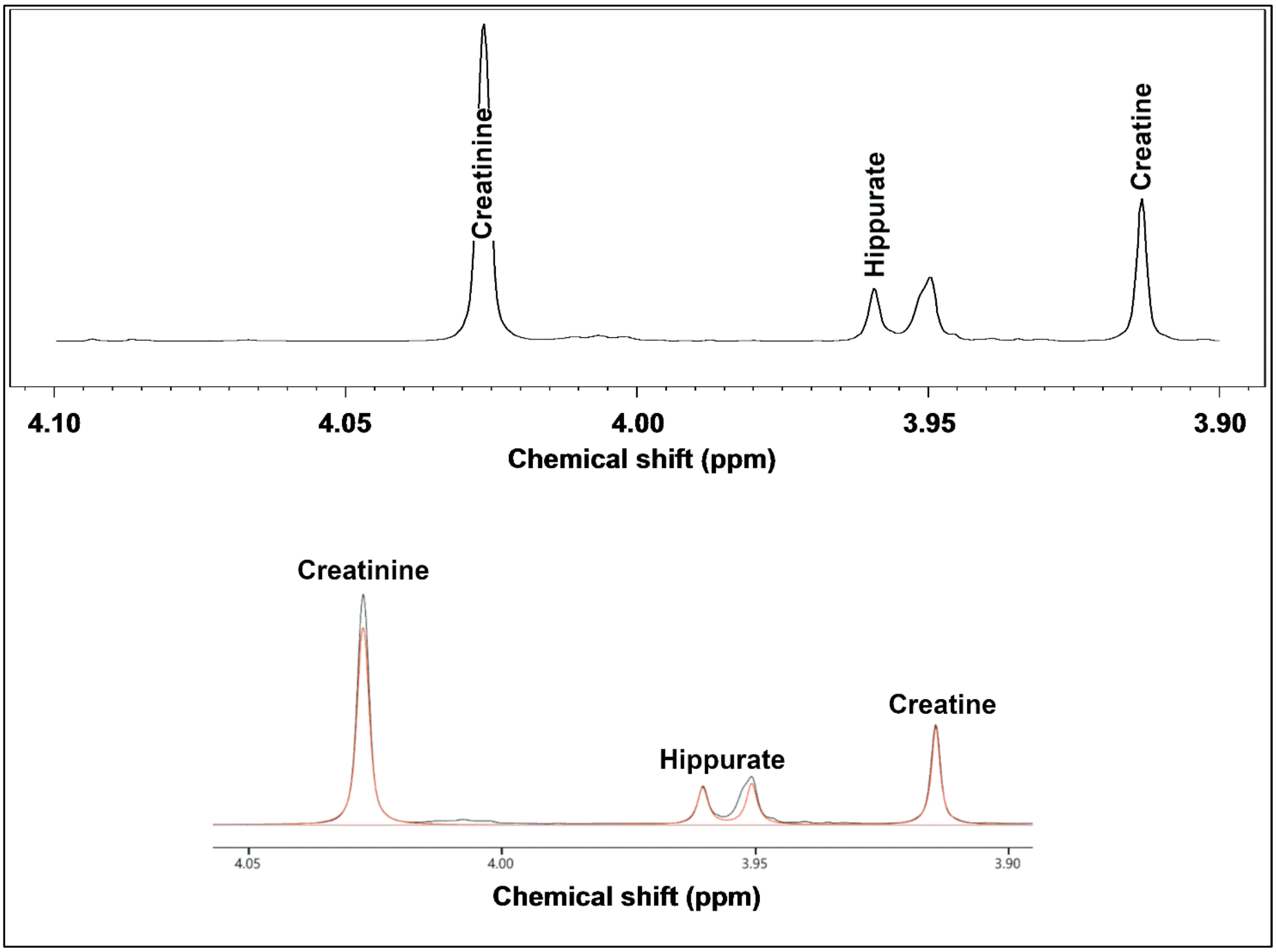
Creatinine
Hippurate
Creatine
4.10
4.05
4.00
3.95
3.90
Chemical shift (ppm)
Creatinine
Hippurate
Creatine
4.05
4.00
3.95
3.90
Chemical shift (ppm)

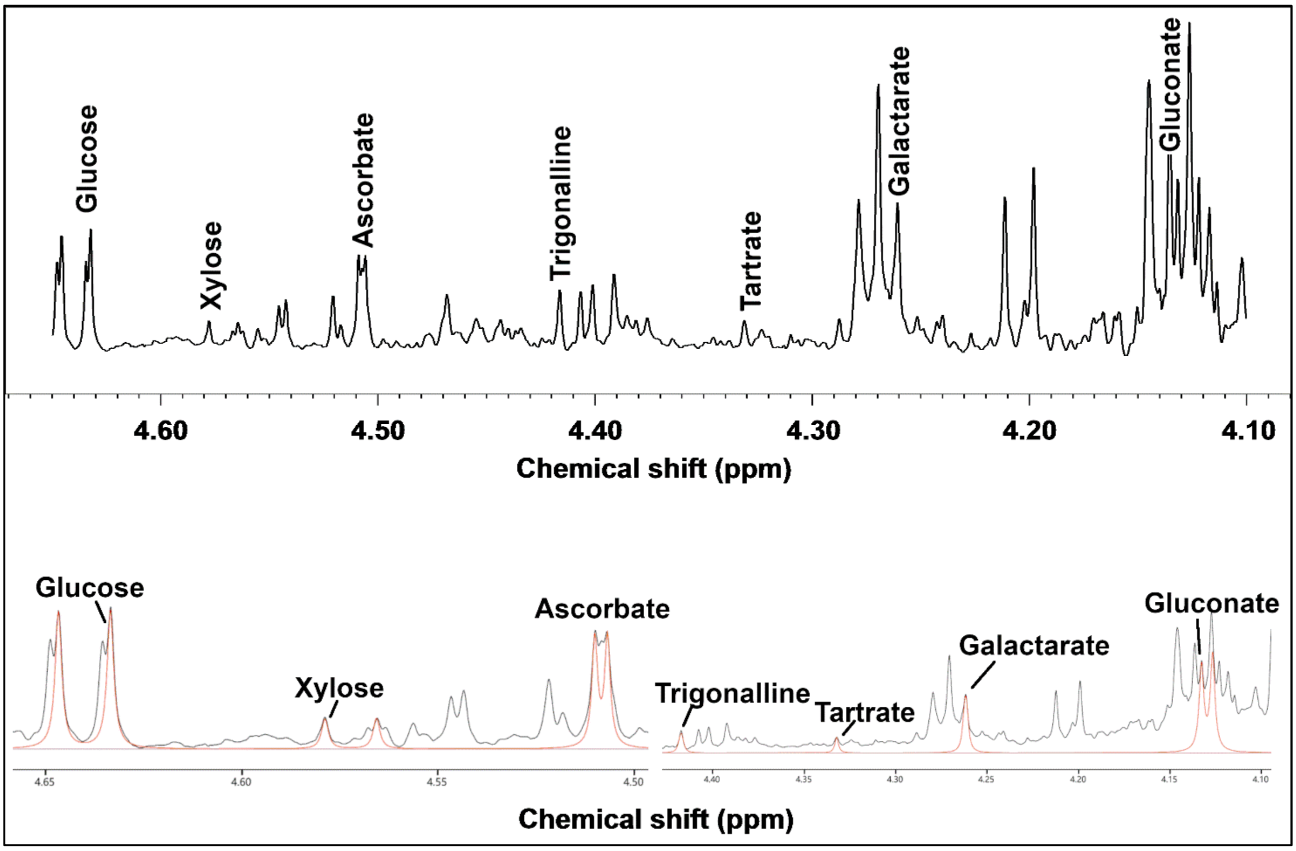
Glucose
Xylose
Ascorbate
Trigonalline
Tartrate
Galactarate
Gluconate
4.60
4.50
4.40
4.30
4.20
4.10
Chemical shift (ppm)
Glucose
Xylose
Ascorbate
Trigonalline
Tartrate
Galactarate
Gluconate
Chemical shift (ppm)

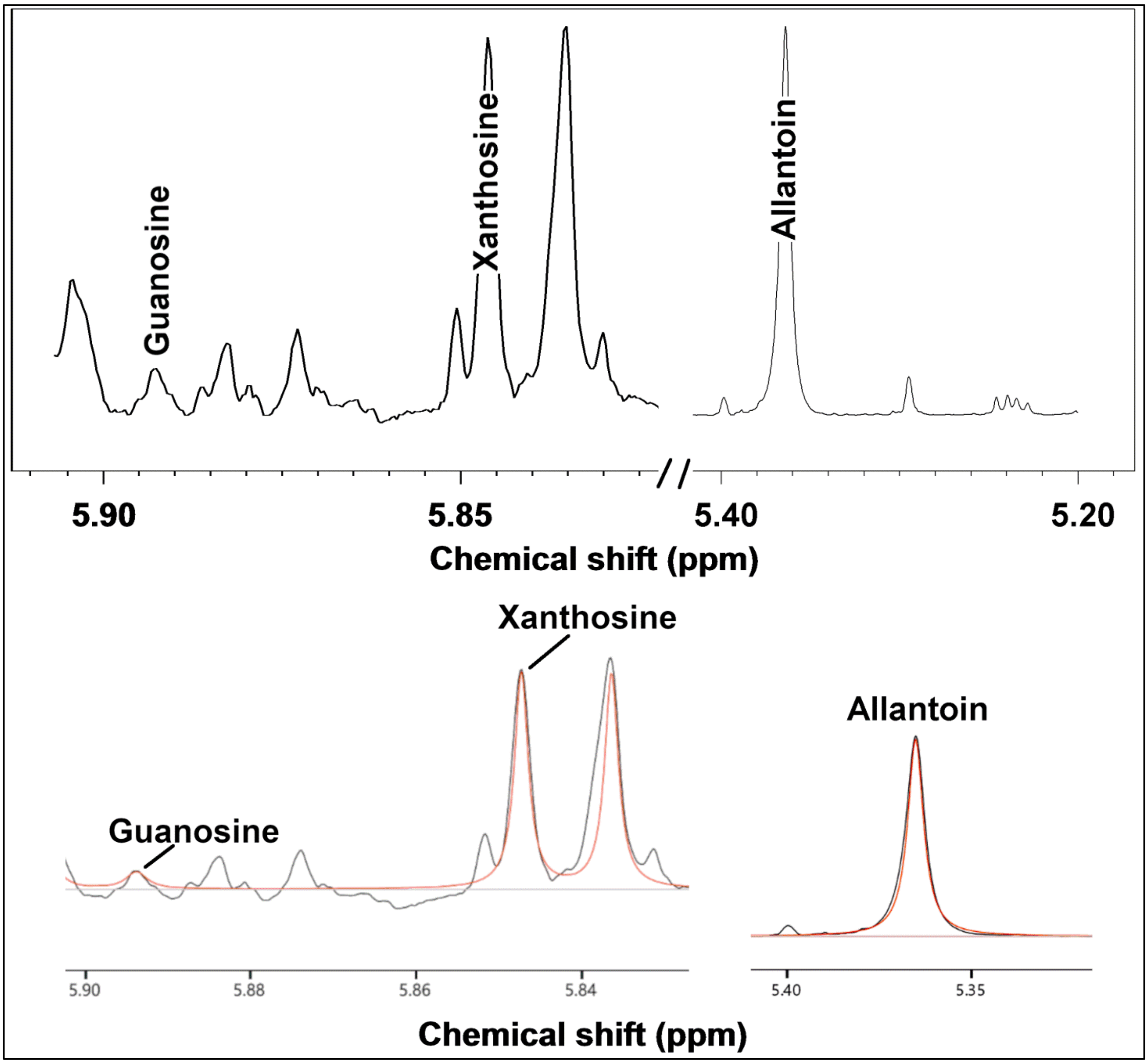
Guanosine
Xanthosine
Allantoin
5.90
5.85
5.40
5.20
Chemical shift (ppm)
Xanthosine
Allantoin
Guanosine
5.90
5.88
5.86
5.84
5.40
5.35
Chemical shift (ppm)

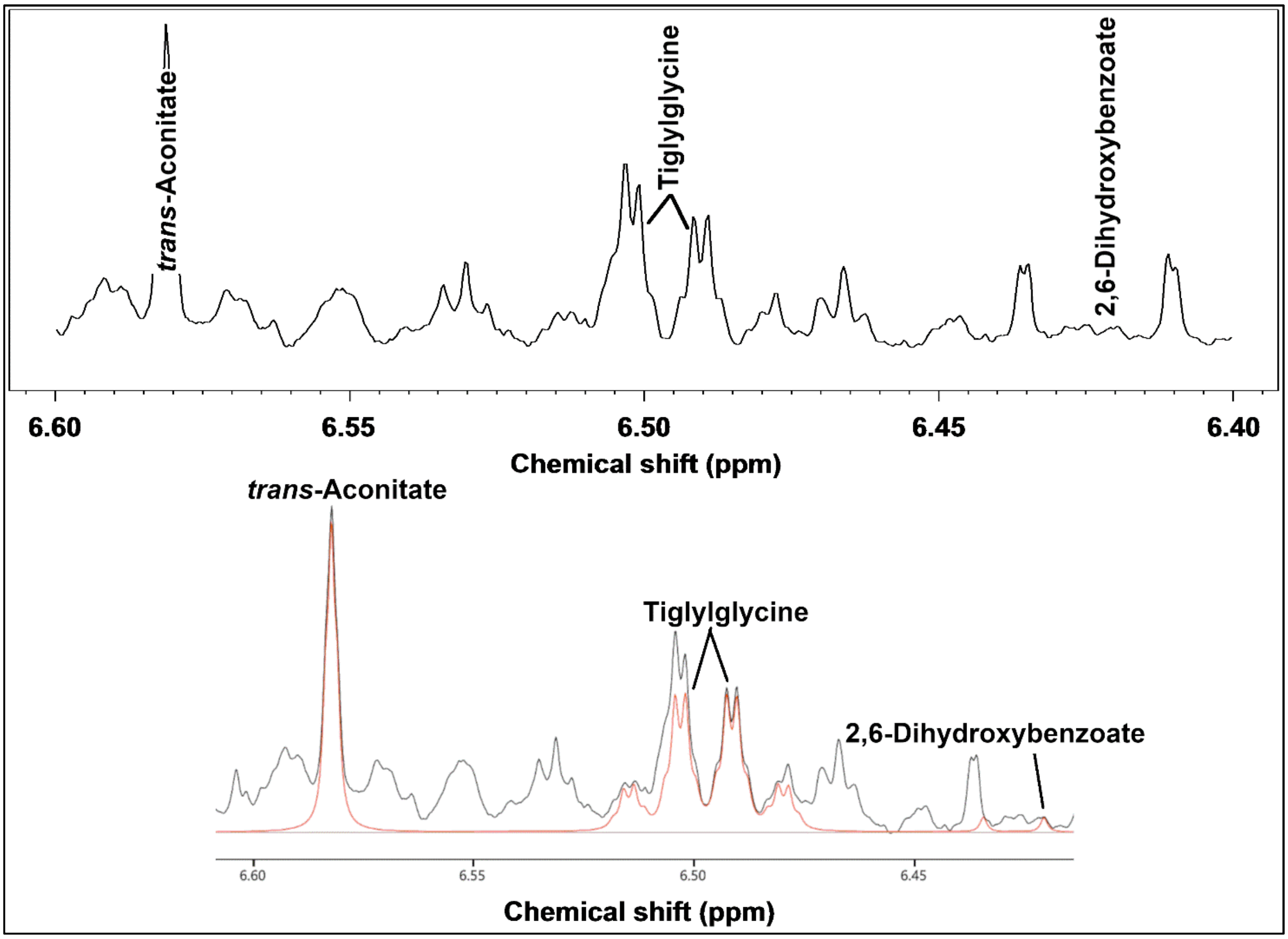
trans-Aconitate
Tiglylglycine
2,6-Dihydroxybenzoate
6.60
6.55
6.50
6.45
6.40
Chemical shift (ppm)
trans-Aconitate
Tiglylglycine
2,6-Dihydroxybenzoate
6.60
6.55
6.50
6.45
Chemical shift (ppm)

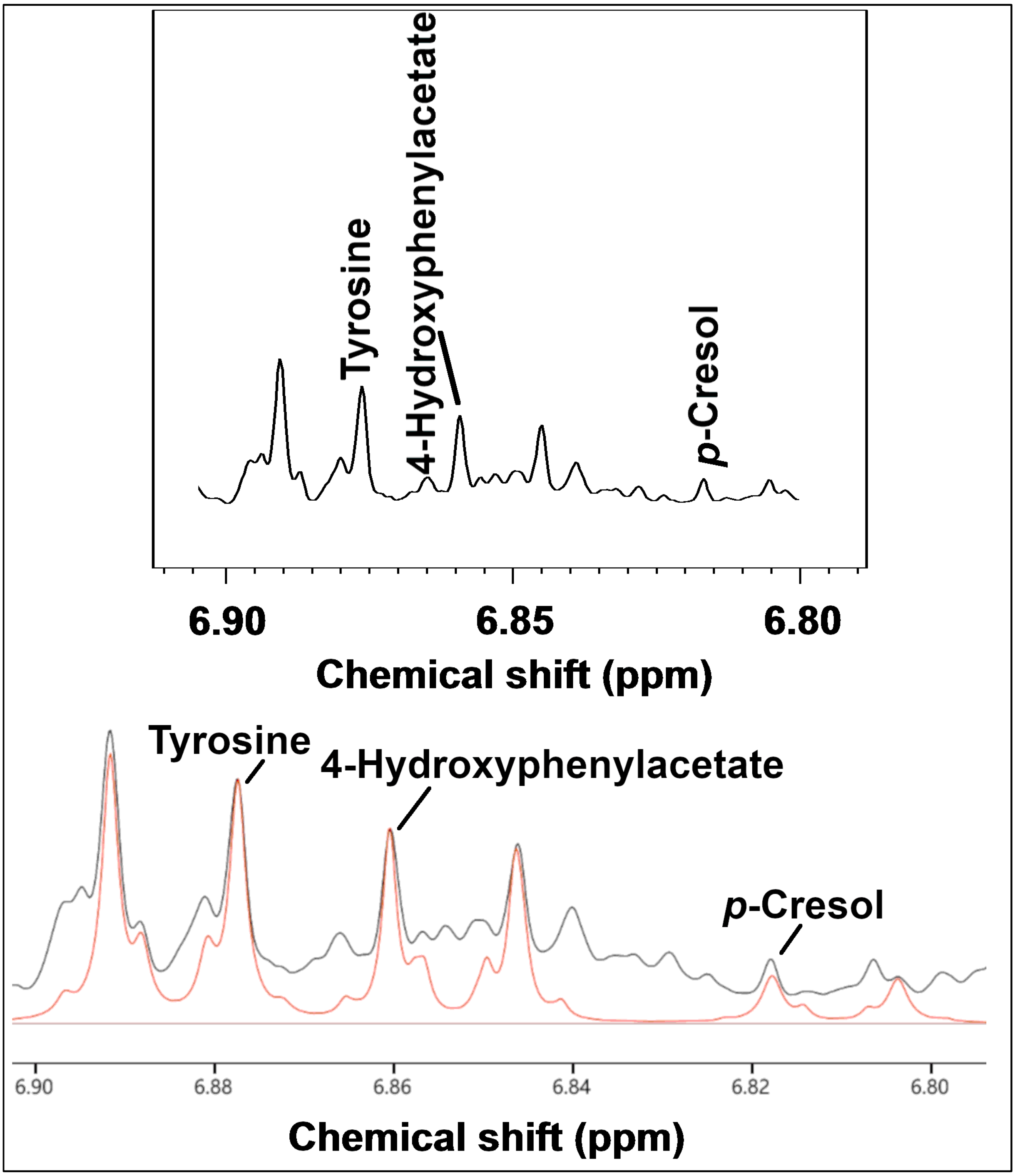

Tyrosine
4-Hydroxyphenylacetate
p-Cresol
6.90
6.85
6.80
Chemical shift (ppm)
Tyrosine
4-Hydroxyphenylacetate
p-Cresol
6.90
6.88
6.86
6.84
6.82
6.80
Chemical shift (ppm)

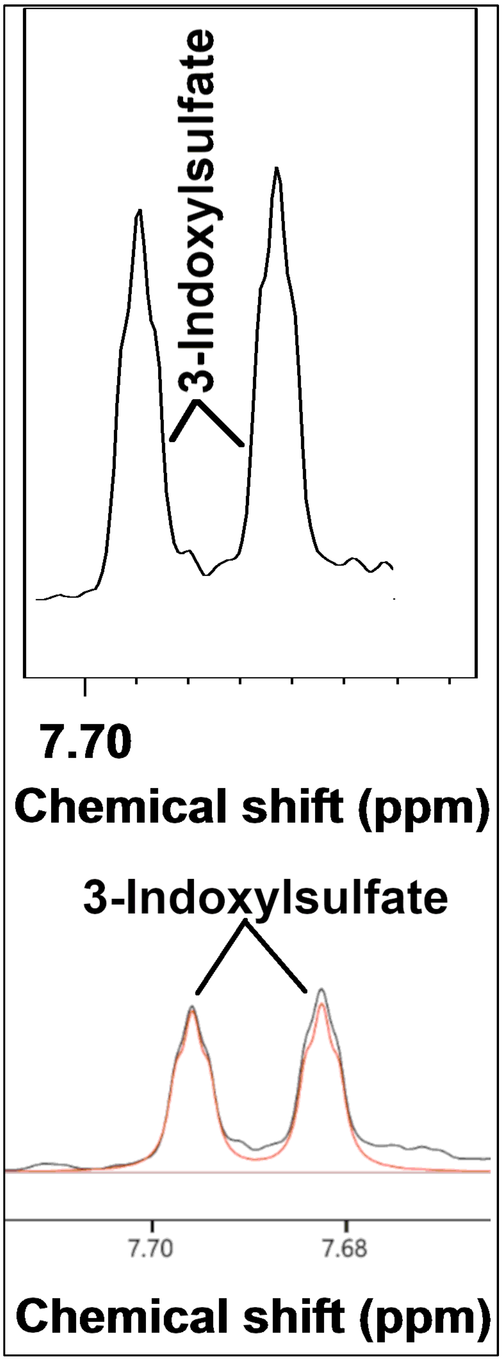
3-Indoxylsulfate
7.70
Chemical shift (ppm)
3-Indoxylsulfate
7.70
7.68
Chemical shift (ppm)

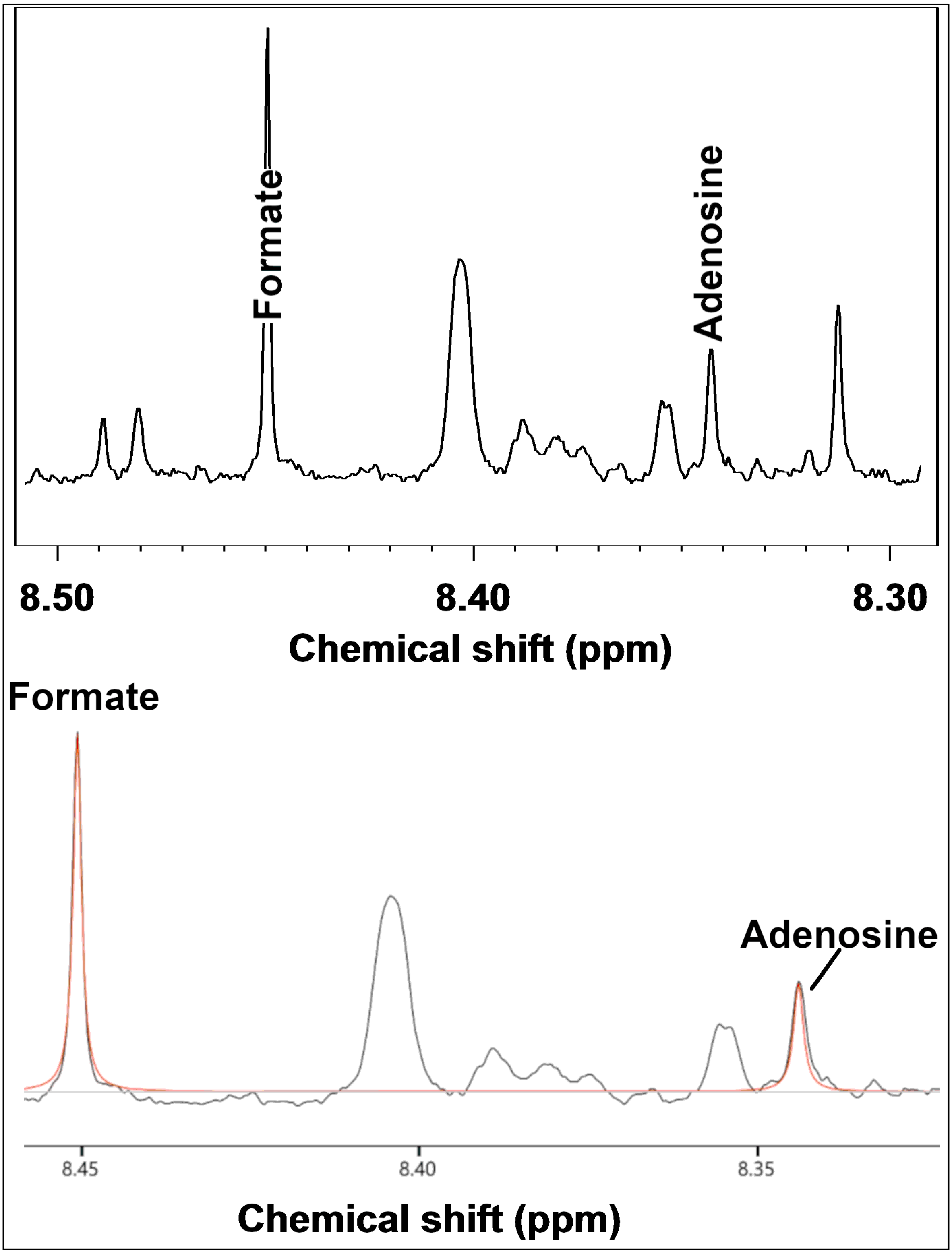
Formate
Adenosine
8.50
8.40
8.30
Chemical shift (ppm)
Formate
Adenosine
8.45
8.40
8.35
Chemical shift (ppm)
